# Automated Purification of DNA Origami with SPRI Beads

**DOI:** 10.1002/smll.202308776

**Published:** 2023-12-06

**Authors:** Chalmers Chau, Gayathri Mohanan, Iain Macaulay, Paolo Actis, Christoph Wälti

**Affiliations:** ^1^ School of Electronic and Electrical Engineering University of Leeds Leeds LS2 9JT UK; ^2^ Bragg Centre for Materials Research University of Leeds Leeds LS2 9JT UK; ^3^ Earlham Institute Norwich Research Park Norwich NR1 7UZ UK; ^4^ School of Biological Sciences University of East Anglia Norwich Norfolk NR4 7TJ UK

**Keywords:** AFM, automation, DNA origami, purification, SPRI beads

## Abstract

DNA origami synthesis is a well‐established technique with wide‐ranging applications. In most cases, the synthesized origami must be purified to remove excess materials such as DNA oligos and other functional molecules. While several purification techniques are routinely used, all have limitations, and cannot be integrated with robotic systems. Here the use of solid‐phase reversible immobilization (SPRI) beads as a scalable, high‐throughput, and automatable method to purify DNA origami is demonstrated. Not only can this method remove unreacted oligos and biomolecules with yields comparable to existing methods while maintaining the high structural integrity of the origami, but it can also be integrated into an automated workflow to purify simultaneously large numbers and quantities of samples. It is envisioned that the SPRI beads purification method will improve the scalability of DNA nanostructures synthesis both for research and commercial applications.

## Graphical abstract

DNA origami is a nanostructure with diverse applications; however, it requires a crucial purification step to remove excess staples before applications. This study introduces the use of solid‐phase reversible immobilization (SPRI) beads as a scalable, high‐throughput, and automatable purification method. This automatable method improves the scalability of DNA nanostructures synthesis for both research and commercial applications.

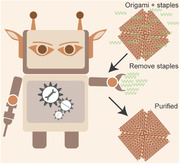

## 1 Introduction

The use of DNA as a building block for the creation of nanoscale materials is the foundation of the field of DNA nanotechnology.^[^ ^1^, ^2^, ^3^, ^4^ ^]^ For example, the DNA origami technique involves the combination of a long ssDNA scaffold with hundreds of short oligonucleotides, “staple” strands, via Watson‐Crick base pairing to assemble rationally designed nanostructures.^[^ ^2^ ^]^ It has found numerous applications in biophysical research, clinical diagnostics, and in cell biology.^[^ ^4^, ^5^, ^6^, ^7^ ^]^ Additionally, the programmability of DNA enables the precise functionalization of the origami nanostructures with a range of biomolecules.^[^ ^4^, ^8^, ^9^, ^10^, ^11^ ^]^ While the assembly and programmability is well‐established in the preparation of such DNA nanostructures, the purification of the desired products from the excess materials used during the assembly, such as staples and biological molecules,^[^ ^3^ ^]^ is still challenging.

A wide range of purification techniques have been developed and are used routinely,^[^ ^12^ ^]^ including gel extraction, poly(ethylene) glycol (PEG) precipitation, molecular weight cut‐off (MWCO) membrane filtration, and spin column‐based filtration.^[^ ^3^, ^4^, ^13^, ^14^ ^]^ Agarose gel electrophoresis separates the slow‐migrating folded DNA nanostructure as a distinct band from the faster migrating staples. The desired band can be excised from the gel and the product extracted.^[^ ^13^ ^]^ An alternative approach relies on the ability of PEG to induce DNA precipitation.^[^ ^14^ ^]^ The third and fourth methods, which are widely used, rely on filtration using MWCO membranes and chromatography resins, respectively.^[^ ^3^ ^]^

**Figure 1:**
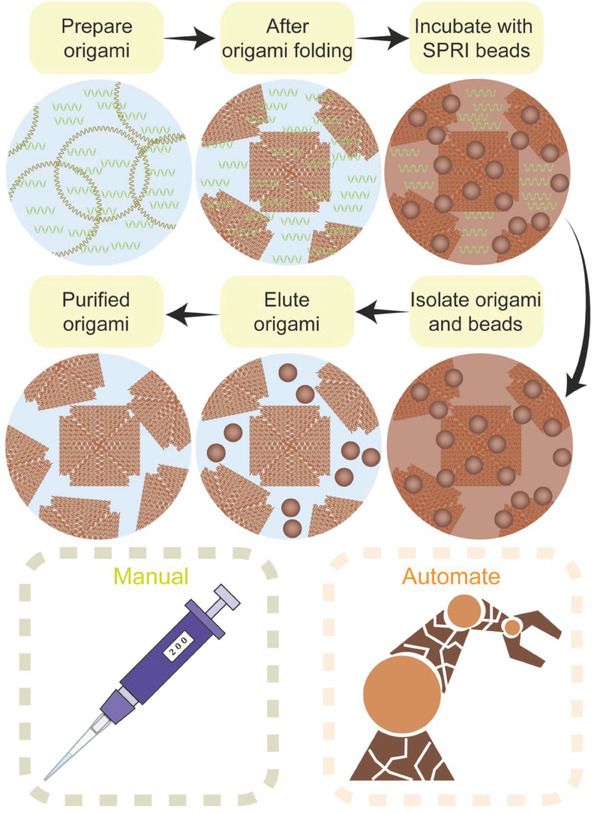
Schematic illustration of SPRI clean‐up of DNA origami. The DNA origamis are mixed with the SPRI beads at a specific beads‐to‐sample volume ratio, followed by their separation with an external magnet. The origami bead pellet is then washed with ethanol followed by elution in the DNA origami storage buffer.

Most of these methods are not suitable for large‐volume purifications and often require manual operations precluding their automation with liquid handling robots.^[^ ^3^ ^]^ This is a major obstacle to the scaling up of their production and implementation in industrial settings. Here, we report the use of solid‐phase reversible immobilization beads (SPRI)^[^ ^15^ ^]^ for the manual as well as automated purification of a range of DNA origami at high concentration from excess staples and proteins (**Figure** **1**; Figure S1, Supporting Information). SPRI beads are paramagnetic microparticles modified with carboxyl groups that can reversibly bind to DNA and are widely employed in transcriptomics for DNA fragment size selection and purification prior to sequencing.^[^ ^15^, ^16^, ^17^, ^18^, ^19^, ^20^ ^]^ In contrast to other methods, this technique does not require centrifugation^[^ ^3^, ^14^, ^21^, ^22^ ^]^ or modifications of the origamis, which present limitations for automated large‐scale implementations, and we demonstrate the readiness of this method for scaling up by performing an automated purification of 96 DNA origami samples with a liquid handling robot.^[^ ^23^ ^]^ We envisage that the SPRI clean‐up method will further the commercial exploitation of DNA nanostructures by enabling their high‐throughput purification.

## 2 Results and Discussion

SPRI beads are widely used in the preparation of DNA sequencing libraries prior to next‐generation sequencing.^[^ ^18^, ^19^, ^20^ ^]^ A typical SPRI beads suspension buffer contains alkali halide salt such as NaCl and high molecular weight PEG, and varying the concentration of salt and PEG will change the size selectivity of the beads.^[^ ^24^, ^25^, ^26^ ^]^ Mixing DNA into the suspension causes the DNA to precipitate onto the carboxyl‐coated microparticles through Ψ condensation. The DNA can subsequently be isolated from the solution with a magnet.^[^ ^26^, ^27^, ^28^, ^29^, ^30^, ^31^, ^32^, ^33^, ^34^ ^]^ To achieve size‐selective separation of DNA, the ratio between the volume of beads and DNA is critical – the smaller the ratio, the longer the size of DNA retained. To demonstrate this, we performed dsDNA size selections on a broad range dsDNA ladder ranging from 75 bp to 20 kbp with different volume ratios of SPRI beads (Figure S2, Supporting Information). When a volume ratio of 0.4X was used, DNA sizes below 1000 bp were removed; in contrast at a volume ratio of 1.0X and above only DNA sizes below 200 bp were removed. Based on this, and the large differences in molecular weight between the excess staples (routinely under 50 nts) and the folded origami, we demonstrate that SPRI clean‐up is an effective method to purify DNA nanostructures from excess staples, and the procedure can also be integrated into existing automated liquid handling robot.

**Figure 2:**
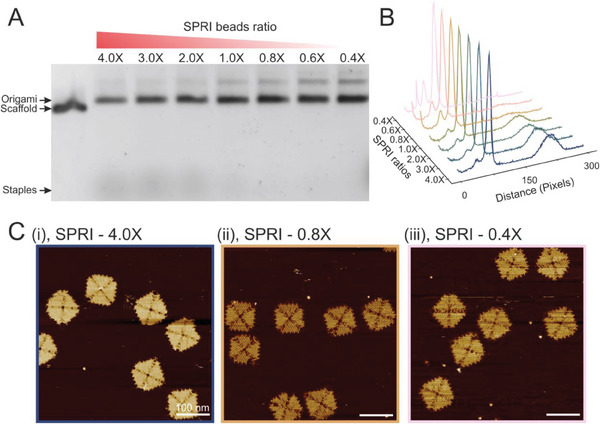
Purification of DNA origami using different ratios of SPRI beads to origami. A), Agarose gel of 4FST origamis after SPRI purification at different volume ratios. B), densitometric profile for all lanes in A. C), AFM images of i), 4X ii), 0.8X iii), 0.4X ratio purified 4FST origami (scale bar:100 nm). All purifications included a thermal de‐clumping step.

An 88 nm × 88 nm fourfold symmetrical tile (4FST) DNA origami^[^ ^35^, ^36^, ^37^ ^]^ was selected and used throughout this study as the model DNA nanostructure for the validation of the SPRI clean‐up. To achieve high‐quality purification, it is important to optimize the volume ratio between the sample and SPRI beads solution.^[^ ^17^, ^24^, ^25^ ^]^ Volume ratios between 0.4X and 4.0X (i.e., for the 0.4X volume ratios, 1 volume of DNA origami solution:0.4 volume of SPRI beads, more details can be found in the supporting information) were investigated using agarose gel electrophoresis to identify the optimal in terms of efficient removal of excess staples from the DNA origami assembly mixture (**Figure** **2**A; Figure S5A, Supporting Information). The folded 4FST origami band migrated slower compared to the scaffold and can clearly be observed as a prominent band in the gel electropherogram. The densitometric lane profile of all volume ratios is shown in Figure 2B. The integrity of the origamis for all ratios were checked with Atomic Force Microscopy (AFM) imaging (Figure 2C; Figures S6–S12, Supporting Information) demonstrating that the SPRI beads did not affect the integrity of the origamis. The total mass of origami in the different bands ranged from ≈700 to ≈1600 ng from a total theoretical maximum of 1880 ng for a 40 µl reaction (Figure S5B, Supporting Information).

It can be seen from the presence of excess staples (fast migrating lower molecular weight materials) in Figure 2A,B that volume ratios of 1X and higher yielded incomplete purification. Ratios of 0.8X or lower resulted in pure origamis with no staples observed in the gel electropherogram. We chose the 0.8X ratio for all downstream testing and successfully purified three other origami designs (dimer 4FST, 4FSF, and frame) to demonstrate that the method is not specific to a single DNA origami design (Figures S13–S16, Supporting Information). The origami structure used here can form higher‐order structures through stacking interactions as shown before,^[^ ^35^, ^38^ ^]^ and the formation of dimers can be observed (Figure 2A).^[^ ^36^, ^39^ ^]^ When the SPRI beads were used at a low volume ratio such as 0.4X, the dimer band became more prominent. This effect could be due to the reduction of the beads surface area as fewer beads are available to interact with the 4FST origamis in the solution.

We note that the SPRI bead‐purified origami clumped together into a cluster (Figure S3, Supporting Information). We hypothesized that the formation of aggregates was the result of the dehydration caused by the ethanol wash during the purification, as previous studies reported that high percentages of alcohol led to the condensation and precipitation of DNA due to electrostatic interactions.^[^ ^40^, ^41^, ^42^ ^]^ Therefore, a thermal de‐clumping step was introduced, which allowed the origami aggregates to be dispersed successfully as evidenced in the agarose gel and AFM images (Figures S3A and S3B, Supporting Information). Alternatively, the tube can be vortexed for a minute (Figure S3C, Supporting Information). Baptist et al. recently observed similar aggregation after purifying origamis via PEG precipitation,^[^ ^14^ ^]^ which they attributed to stacking interactions between origamis after prolonged centrifugation.^[^ ^43^ ^]^ Although no centrifugation was involved with our SPRI clean‐up method, stacking interactions could also have been enhanced during the ethanol wash step. Importantly, the thermal de‐clumping does not lead to degradation of the origami as evidenced by the AFM images in Figure 2A. In addition to the SRPI beads used here, other commercially available SPRI beads were tested, and both successfully purified origami (Figure S4A, Supporting Information). Several studies have shown that the SPRI beads buffer can be adjusted and substituted with other buffers.^[^ ^24^, ^25^ ^]^ We replaced the SPRI beads buffer with a custom‐made buffer composed of PEG, NaCl, and TE and found that the performance of the SPRI purification of DNA origamis is unaffected (Figure S4B, Supporting Information), opening the approach to applications where specific buffer conditions are required.

**Figure 3:**
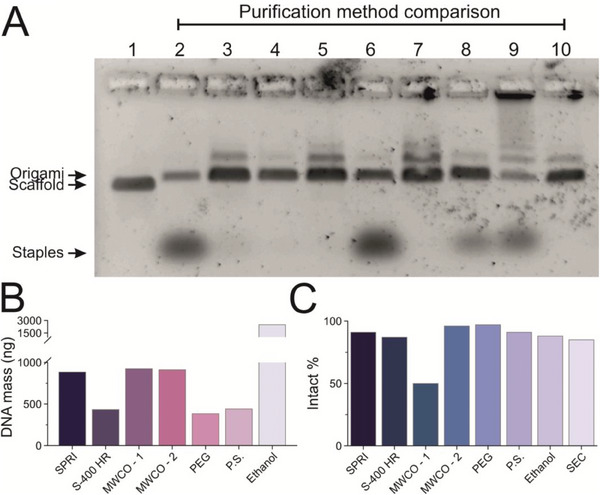
Comparison of different methods to purify 4FST origami. A), Agarose gel image of origami purified using different methods. Lane: 1, M13mp18 scaffold; 2, uncleaned 4FST tile; 3, 0.8X SPRI volume ratio; 4, S‐400 HR spin column; 5, 100 kDa MWCO‐1 filter; 6, 100 kDa MWCO‐2 filter; 7, PEG precipitation (PEG); 8, Phase separation (P.S.); 9, Ethanol precipitation (Ethanol); 10, Size‐exclusion column (SEC) purified. A total mass of 25 ng was loaded in each lane, a calculation based on A260 UV absorption measurements. B), Purification yield calculated from A260 absorption measurements. The unexpectedly high DNA mass for ethanol purification could be due to excessive clumping. SEC data were not included, as the preparation of the SEC samples had to be carried out with 8 times the amount of staring material than other methods, and it included the use of a MWCO concentrator to increase the concentration of the eluted origami samples. C), Percentage of intact origami after purification for each method quantified by AFM imaging. A detailed summary can be found in Table S1 and Figure S2 (Supporting Information).

Various DNA origami purification techniques have been reported before^[^ ^12^ ^]^ and we selected, to the best of our knowledge, the most widely used methods to perform a systematic study to benchmark the SPRI bead purification approach. These methods include the S‐400 HR spin column filtration, two different 100 kDa MWCO filtrations, PEG precipitation, phase separation, ethanol precipitation, and size exclusion chromatography (SEC).^[^ ^3^, ^14^, ^21^, ^22^, ^44^ ^]^ The uncleaned origami samples and origami purified using each method were analyzed via agarose gel electrophoresis (**Figure** **3**A). The uncleaned sample showed a well‐defined band at the molecular weight expected for the DNA origami, as well as a broad and intense band corresponding to low molecular weight products that were attributed to the excess staples. Judging from the gel electropherogram (Figure 3A), all methods, except the 100 kDa MWCO‐2 (lane 6), phase separation (lane 8), and ethanol precipitation (lane 9) successfully purified the DNA origami from the excess staples. For the ethanol precipitation method, a bright band can be seen in the well, similar to what we had observed in SPRI clean‐up prior to the thermal de‐clumping (Figure S3, Supporting Information), suggesting that significant clumping occurred.

When the total DNA origami purification yield was measured (Figure 3B), the ethanol precipitation method delivered the highest yield followed by the two MWCO methods, but importantly, a significant number of staples were still present in the MWCO‐2, phase separation, and ethanol precipitation sample following purification which contributed to the overall yield (Figure 3A,B). The SPRI purification yielded a similar amount of purified DNA origami as MWCO‐1 and MWCO‐2. Lastly, we assessed the structural integrity of the origamis following purification for each of the methods discussed above by AFM (Figures S17 and S23 and Table S1, Supporting Information). All purifications but MWCO‐1 retained 80% or more of the origami structurally intact (Figure 3C). For MWCO‐1, significant deformation of the 4FST structure could be observed (Figure S18, Supporting Information). Overall, the SPRI bead purification method is either the best‐performing or at least in line with the best‐performing techniques in terms of yield, DNA origami purity, and structural integrity.

**Figure 4:**
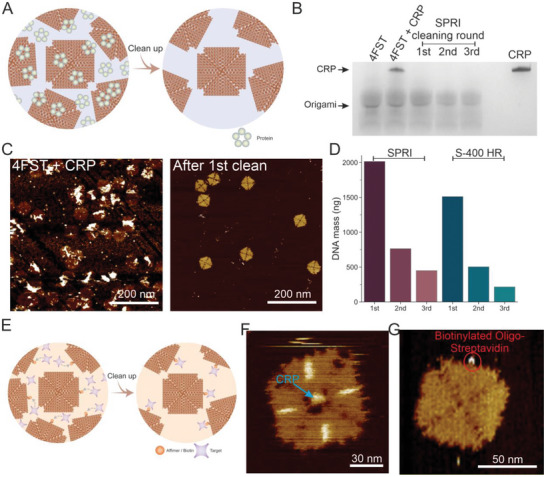
SPRI assisted removal of excess proteins. A), Schematic illustration of removing excess proteins from DNA origami. B), Silver‐stained denaturing SDS‐PAGE gel showing CRP–DNA origami sample after one, two, and three rounds of SPRI bead purification, together with cleaned and uncleaned origami, and CRP as controls. C), AFM images of the CRP–DNA origami sample before (left) and after the 1st round of SPRI cleaning (right; scale bar: 200 nm). D), Yield by DNA mass of origamis after three rounds of cleaning using 0.8X volume ratio SPRI bead and S‐400 spin columns. E), Schematic illustration of streptavidin conjugation to DNA origami containing biotinylated staples and the subsequent removal of excess streptavidin using a 0.8X volume ratio of SPRI beads. F), AFM image of CRP bound to a CRP‐affimer functionalized 4FSF frame. The CRP is indicated by the blue arrow. G), AFM image of streptavidin conjugated to a biotinylated 4FST tile. The streptavidin bound to the biotinylated staple is indicated by a red circle.

For a wide range of applications, DNA origami has to be functionalized with fluorophores, proteins, and nanoparticles, inter alia.^[^ ^4^, ^8^, ^9^, ^10^, ^11^, ^45^ ^]^ To achieve high functionalization yields, the functionalization materials are added to the DNA origami in significant excess, and therefore this excess material needs to be removed during purification to prevent interference with downstream applications while retaining the functionalized DNA origami.^[^ ^46^ ^]^ SPRI bead clean‐up is not only an effective method to purify DNA nanostructures from excess DNA staples but can also be employed to remove functionalization materials such as proteins. Here we used C‐reactive protein (CRP) as a model system to demonstrate the effectiveness of the SPRI bead purification as it has a considerable size (125 kDa) which proves challenging for conventional purification methods such as membrane purification.^[^ ^4^, ^46^ ^]^ CRP was added to the SPRI bead‐purified 4FST origami at a 2:1 molar ratio, and the mixture was then cleaned using the same volume ratio of 0.8X of SPRI beads as for removing the staples after the origami assembly (**Figure** **4**). The DNA origami‐CRP mixture was SPRI bead cleaned multiple times in succession to ensure the complete removal of CRP as well as to investigate the effect of successive purification rounds. Surprisingly, we observed that CRP was completely removed during the first cleaning step as can be seen by SDS‐PAGE gel in Figure 4B, where the band corresponding to CRP disappeared. This effective purification was confirmed by AFM where the image is dominated by CRP in the background before the SPRI bead clean‐up (Figure 4C, left). After the purification (Figure 4C, right), the background is clean and the DNA origami is predominantly intact. AFM images of the DNA CRP–DNA origami sample after all purification rounds are shown in Figure S24 (Supporting Information). Furthermore, we assessed the performance of the successive SPRI purifications in terms of retaining DNA origami at different SPRI bead volume ratios and using S‐400 HR spin column purification for comparison (Figure 4D; Figures S25–S27, Supporting Information). We found that SPRI bead purification retained a high proportion of structurally mostly intact DNA origami structures even after multiple rounds of clean‐up with ≈500 ng left after three rounds, in contrast to only ≈200 ng left after three rounds of S‐400 HR spin column purification. These findings clearly demonstrate the power of SPRI bead purification to remove effectively excess functionalization material from DNA origami with high yield and little structural impact.

In scenarios where excess materials are used to functionalize the DNA origami, it's important that the purification method does not interfere with or reverse the functionalization (Figure 4E). To test this, we investigated the functionalization of DNA origami with two different types of molecular interactions: CRP affimer to CRP (Figure 4F) and biotin to streptavidin (Figure 4G).

Affimers are small monomeric protein scaffolds based on a consensus sequence derived from cystatin proteases containing two variable peptide loops for target interactions.^[^ ^47^, ^48^ ^]^ The variable loops can be tailored through phage display technology to turn the affimers into target‐specific binding molecules,^[^ ^47^, ^49^, ^50^, ^51^ ^]^ and have been employed in immune‐affinity assays and biosensors for target recognition.^[^ ^50^, ^52^ ^]^ Here, we used SPRI beads to purify 4FSF DNA origami functionalized with CRP‐specific affimers through click‐chemistry (Figure S28, Supporting Information) and DNA hybridization (see Supporting Information for Experimental Section).^[^ ^50^ ^]^ The reaction mixture was subjected to the thermal de‐clumping procedure, followed by incubation of CRP at a molar ratio of 18:1. Subsequently, SPRI bead clean‐up was used to remove the excess CRP from the mixture. The binding of CRP to the affimer to the center of the 4FSF frame as intended was confirmed via high‐resolution AFM (Figure 4F). Importantly, during this process, the affimer was subjected to a thermal step twice and retained its ability to bind CRP, demonstrating the SPRI clean‐up procedure does not alter the functional properties of the affimer.

We also investigated the ability of the SPRI clean‐up method to purify a different class of functional DNA origami. The 4FST DNA origami was functionalized with streptavidin through interaction with a biotinylated staple strand^[^ ^4^, ^8^, ^9^, ^10^, ^11^ ^]^ (Figure 4E,F). We employed SPRI bead clean‐up alongside the S‐400 HR spin column filtration on the origami after incubating the biotinylated origami with streptavidin at 2:1 molar ratio. The percentage of still intact streptavidin functionalization, that is, the percentage of origami that retained the streptavidin, after purification, was quantified by AFM (Figure S30C, Supporting Information). We found that SPRI purification led to a high percentage (≈85%) of streptavidin being retained on the origami compared to S‐400 HR filtration where only about half of the origami had streptavidin bound after the clean‐up, demonstrating the excellent performance and advantage of SPRI bead purification for applications where functionalized origami is required.

**Figure 5:**
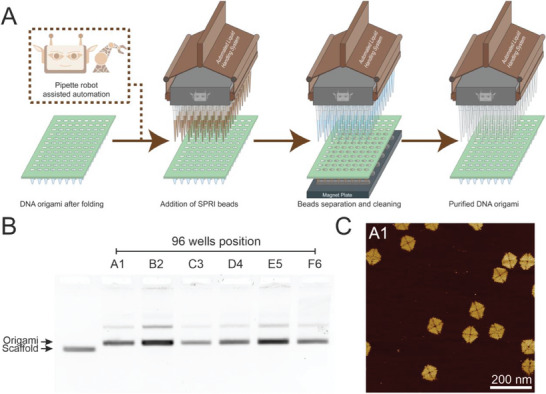
Automated robotic SPRI bead purification of 4FST origami. A), Schematic illustration of the liquid handling robot performing the SPRI purification procedure on the origami samples. B), Agarose gel electrophoresis analysis of six samples of the DNA origami purified via automation. C, AFM image of a sample from well A1 (scale bar:200 nm).

The key advantage of SPRI bead‐based purification is that it is compatible with large‐scale robotic purification. Here, we demonstrate that the SPRI clean‐up can be automated by implementing the protocol on an automated liquid handling robot, which enables the purification of DNA origami from 96 reactions simultaneously within 30 min (**Figure** **5**A). Following the folding reaction inside a 96‐well PCR plate, the liquid handling robot was programmed to perform the SPRI purification procedure, and after the elution of the origami, the plate was transferred to the thermocycler for the thermal de‐clumping step. The purification procedure was carried out with two different SPRI beads, HighPrep SPRI beads and SPRIselect beads. All purified products were analyzed via agarose gel electrophoresis (Figure 5B; Figures S31 and S32, Supporting Information) and a selection by AFM imaging (Figure 5C; Figure S33, Supporting Information). For both 96 parallel robotic purification procedures, bands showing the presence of DNA origami were observed in 95 out of the 96 purification procedures. AFM images of six purified products demonstrated that the robotic purification has minimal structural impact of the DNA origami analogous to the manual purification procedure. These results demonstrate that the automated robotic purification performs as well as the manual procedure.

The SPRI bead purification procedure requires only basic laboratory instruments, reaction tubes, pipettes, and magnets,^[^ ^15^, ^16^ ^]^ thus, this procedure is directly compatible with automation where minimal manual interference is required.^[^ ^23^ ^]^ In contrast, methods that are based on precipitation or filtration rely on centrifugation^[^ ^3^, ^14^, ^21^, ^22^ ^]^ which is a significant obstacle for automation. Besides the automated liquid handling robot we used here (Biomek NX^P^ Automated Workstation), various models of automated liquid handling robots can be found on the market that provide either full automation or partial automation including the chemagic Prepito from Revvity, KingFisher from Thermo Fisher Scientific, and Assist Plus from Integra, alternatively, liquid handling machines can be made with custom 3D printed parts.^[^ ^53^, ^54^ ^]^ Upgrading to robotic purification allows the clean‐up of large volumes of DNA origami time efficiently and provides high throughput, as well as making it more reproducible and less error‐prone. Automation is key in achieving scalability as it can handle large quantities of samples, and additionally, post‐processing such as lyophilization or cryo‐preservation can be implemented after the SPRI purification to store and transport purified DNA nanostructures at large quantities,^[^ ^43^, ^55^, ^56^ ^]^ which can be critical for enabling commercial exploitation of DNA nanostructures.

Magnetic beads have been used previously to purify origami, but the reported approach relies on the chemical coupling of the DNA origami to the surface of the magnetic beads.^[^ ^44^, ^57^ ^]^ The SPRI clean‐up selectively removes short oligos by varying the beads volume ratio (Figure S2, Supporting Information), and the significant differences in molecular weight between the folded origami and staples suggest that the SPRI clean‐up can be universally applied to any DNA origami design, irrespective of the oligo or functional molecule used. Previous studies have suggested that the buffer component of the SPRI beads could be further refined to increase the size‐selectivity of the beads,^[^ ^24^, ^25^ ^]^ and thus we anticipate that the composition of the SPRI mixture can be optimized in future to further enhance the clean‐up efficiency.

## 3 Conclusion

We have demonstrated the use of SPRI beads, which are well‐established in sequencing, as an innovative technique to purify DNA origami with high yield and high structural integrity. We analyzed the efficiency of this technique at different volume ratios of beads to DNA origami and selected the optimum ratio for purification. This method is universal and can be applied for wide range of origami designs. We showed that the SPRI technique can be used as a reliable method with comparable yields to existing purification methods. Moreover, we demonstrated the use of this method to remove excess materials used to functionalize the DNA origami, such as proteins, and found that a high proportion of the origami was still fully functionalized after the purification, while excess materials used for the functionalization was removed with high yield. Lastly, we demonstrated the scalability of the purification technique, that is, the possibility of expanding origami purification from the lab bench to an industrial process, by automating the SPRI bead clean‐up procedure. Successful implementation of high‐throughput automation to prepare purified origami of various designs means increased scalability and adaptability not only for research but also for relevant industry sectors, assisting the development of DNA nanotechnology and its successful commercialization.

## 4 Experimental Section

For Experimental Section details, please refer to the Supporting Information.

## Conflict of Interest

The authors declare no conflict of interest.

## Author Contributions

C.C. and G.M. contributed equally to this work. C.C. designed the research study, and G.M. performed the experiments. C.C. and G.M. analyzed data. C.C. illustrated all schematics. I.M. helped with the robot automation. P.A. and C.W. supported the data analysis, supervised the work, and acquired the funding. All authors contributed to the writing of the manuscript.

## Supporting information

Supporting Information

## Data Availability

The data that support the findings of this study are openly available in [University of Leeds] at [https://doi.org/10.5518/1369], reference number [1369].
