# Supplementary material for: Automated Purification of DNA Origami with SPRI Beads
Source: Small. 2023 Dec 6;20(20):2308776. doi: 10.1002/smll.202308776 (PMC11475516; doi:10.1002/smll.202308776)
Supplement: Supplementary file 1 — Supporting Information [file SMLL-20-2308776-s001.pdf]

NANO · MICRO
small

Supporting Information

for *Small*, DOI 10.1002/smll.202308776

Automated Purification of DNA Origami with SPRI Beads

Chalmers Chau, Gayathri Mohanan, Iain Macaulay, Paolo Actis and Christoph Wälti**

Supporting Information for:

Automated Purification of DNA Origami with SPRI Beads

Chalmers Chau[1,2] *ǂ, Gayathri Mohanan[1,2]ǂ, Iain Macaulay[3,4], Paolo Actis[1,2], Christoph Wälti[1,2] *

[1] School of Electronic and Electrical Engineering, University of Leeds, LS2 9JT, UK

[2] Bragg Centre for Materials Research, University of Leeds, LS2 9JT, UK

[3] Earlham Institute, Norwich Research Park, Norwich, NR1 7UZ, UK

[4] School of Biological Sciences, University of East Anglia, Norwich, Norfolk, NR4 7TJ, UK

ǂ These authors contribute equally.

* Corresponding: c.c.chau@leeds.ac.uk; c.walti@leeds.ac.uk;

Table of Contents

Table of Contents 2

Section 1: Supporting Methods 4

DNA origami production 4

4-Fold Symmetrical Tile (4FST) and the biotinylated 4FST origami 4

Dimeric 4-Fold Symmetrical Tile (4FST) 5

4-Fold Symmetrical Frame (4FSF) and CRP-affimer functionalised 4FSF origami 5

Frame origami 6

DNA origami purification 6

SPRI beads selection 6

S-400 HR spin column filtration 7

Molecular weight cut-off (MWCO) membrane filtration 8

PEG precipitation 8

Phase separation 9

Ethanol precipitation 9

Size Exclusion Chromatography (SEC) 9

Automated purification via liquid handling robot 10

DNA origami yield measurement 10

Absorption spectroscopy 10

Fluorescence 10

Size selection of the DNA ladder 11

Agarose Gel analysis 11

Origami-protein mixture clean-up 12

SDS-PAGE analysis 12

Atomic Force Microscopy (AFM) 12

Intact origami percentage calculation 13

Streptavidin functionalised origami percentage calculation 13

CRP bound percentage of the CRP affimer functionalised origami calculation 14

Section 2: Supporting Figures 15

Section 3: Supporting Instruction 43

References 44

Section 1: Supporting Methods

All chemicals were acquired from Sigma-Aldrich at ACS grade unless otherwise stated. All concentrations stated are the final concentration unless otherwise stated. All buffers (except buffers containing PEG or Dextran) were sterile filtered with either 0.1µm (16553K; Sartorius) or 0.22µm (SLGP033RS; Millipore) syringe filters.

DNA origami production

All DNA strands used throughout this study were purchased from Integrated DNA Technologies (IDT) with standard desalting. The caDNAno (2.4.7) software was used to design the DNA origami structures [1]. All origamis were folded using the 7249 nt M13mp18 circular single-stranded DNA scaffold (N4040S; NEB). All folded origamis were stored at 4°C or immediately purified.

The caDNAno files of the origami designs, list of staples and the M13mp18 sequence are included in the repository associated with this publication.

4-Fold Symmetrical Tile (4FST) and the biotinylated 4FST origami

The design of the 4-Fold Symmetrical Tile (4FST) DNA origami used here follows the design published by Tikhomirov *et al.* [2]. The 4FST DNA origami staples are categorized into 4 staple mixtures: interior, bridge, edge and negation, each mixture is a mixture of all relevant staples.

To fold 40 µl of the 4FST DNA origami, the folding mixture contained: 10 nM M13mp18 scaffold, 12.5 mM magnesium acetate, 75 nM bridge mixture, 75 nM interior mixture, 75 nM edge mixture, and diluted in 10 mM tris-HCl, 1mM Ethylenediaminetetraacetic acid (EDTA) at pH 8.0 (1X TE buffer) (93283; Sigma-Aldrich). The folding of the 4FST DNA origami was carried out with a thermal cycler by heating the folding mixture to 90°C for 2 minutes, followed by a gradual temperature decrease from 90°C to 20°C at the rate of 1°C per minute. The temperature was then further decreased to 4°C until use.

For the biotinylated 4FST DNA origami, one of the standard edge staples (Edg-T1R10C7-DHP) in the edge staple mixture was replaced with a 5' biotinylated version of the same staple. To fold the biotinylated 4FST, the same procedure as the standard 4FST was used. After the purification of the biotinylated 4FST origami, a total of 20 nM streptavidin (434301; Thermo Fisher) was added to the 4FST origami and incubated for 10 minutes at room temperature, then the biotinylated 4FST and streptavidin mixture was subjected to purification again.

Dimeric 4-Fold Symmetrical Tile (4FST)

To generate the dimer 4FST, the original set of edge staples were replaced with a different set of edge staples that allows the hybridisation between two 4FST [2, 3]. After the folding of the two separate 4FST origamis, the two samples were mixed at 1:1 volume (including all excess staples). The mixture was then heated to 55°C and the temperature was then gradually decreased to 20°C at a 1°C/min rate. The temperature was then further decreased down to 4°C until use.

4-Fold Symmetrical Frame (4FSF) and CRP-affimer functionalised 4FSF origami

The basic design principle of the 4-Fold Symmetrical Frame follows the 4FST, thus the folding mixture components and the steps were the same as 4FST except for the differences in staple sequences.

The functionalisation of the 4FSF origami involves two steps: conjugation of an affimer to an oligonucleotide and hybridisation of the affimer-oligonucleotide complex into the 4FSF.

For the first step, the 5' end of an oligonucelotide with the sequence of 5'-TCTACTATGGCGGGTGATA-3' was modified with maleimide (Biomers). The previously established CRP affimer with a single cysteine and his-tagged was conjugated to the maleimide oligos through click reactions, the reaction was carried out by mixing 0.1nM of the maleimide oligos with 38μM affimer in 1X TE buffer, pH 8.0 at room temperature for 2 hours. After the conjugation, excess oligos were removed through the Ni-NTA resin, the conjugated samples were mixed with Amintra Ni-NTA affinity resin (ab270549; Abcam) in a volume ratio of 5:1 (resin to conjugate). The mixture was incubated for 2 hours at room temperature under agitation, followed by centrifugation at 1000g for 1 minute to pellet the Ni-NTA resin and remove the supernatant. A wash buffer (50mM monosodium phosphate, 500mM sodium chloride, 30mM imidazole at pH 7.4) was used to rinse the assembled 2 ml disposable columns (29920; Thermo Fisher). 1 ml of the wash buffer was used to resuspend the Ni-NTA resin pellet and transfer to the disposable columns. The wash buffer flowed through the resin via gravity and multiple washing steps were performed until the A_{280} absorbance reading dropped under 0.09. The resin inside the column was then resuspended with the elution buffer (50mM monosodium phosphate, 500mM sodium chloride, 300mM imidazole and 20% glycerol at pH 7.4) and incubated for 5 mins inside the column (capped to prevent elution). The eluate was collected in tubes and monitored by A_{280}, the same elution step was repeated by adding more elution buffer to the resin until the A_{280} absorbance reading dropped to under 0.09. The eluted affimer oligo conjugate was then buffer exchanged to 1X TE, pH 8.0 by Zeba spin desalting columns (89882; Thermo Fisher). The conjugated affimer oligo was snap-frozen and then stored

at -80°C in 1X TE, pH 8.0 at 2.5µM. SDS-PAGE was used to confirm the attachment of the affimer to the oligos. The production of the affimer can be found in previously established works [4].

For the second step, to allow the hybridisation between affimer oligos and the 4FSF origami, 4 staples (4FSF_IEdg-T1L1R13C2-DHP, 4FSF_IEdg-T2L1R13C2-DHP, 4FSF_IEdg-T3L1R13C2-DHP, 4FSF_IEdg-T4L1R13C2-DHP) were replaced so that the 3'end of these staples contained complementary sequences to the affimer attached oligos (5'-TATCACCCGCCATAGTAGA-3'). The 4FSF was folded as outlined above and purified through the SPRI beads selection method at volume ratio of 0.8X (see the DNA origami purification section for the method). Before the thermal declumping step, the 2.5µM affimer oligo was added to the eluted DNA origami solution at 15 nM so that it reached a final concentration of 140nM of affimer oligos. A modified thermal declumping step was used to simultaneously de-clump and hybridise the affimer oligo to the 4FSF origamis (heated to 50°C for 5 minutes, followed by gradual decrease to 20°C at the rate of 0.5°C per minute). The resultant DNA origamis were then incubated with 280nM of C-reactive proteins (CRP; 140-11R; Lee-Bio) for 1 hour to facilitate the binding of CRP to the affimer. The mixture was purified again with the SPRI beads selection method at a volume ratio of 0.8X.

Frame origami

The design of the frame origami used here followed a previously published design by Raveendran *et al.* [5]. All the staples were pooled together into the staple mixture. The origami folding mixture contains 10 nM M13mp18 scaffold, 50 nM staple mixture, 5 mM tris acetate (pH 7.4), 5 mM magnesium acetate and 0.5 mM EDTA. The folding of the frame origami was carried out by heating the mixture to 95°C for 5 minutes followed by gradual temperature decrease to 20°C. The temperature was then further decreased down to 4°C until use.

DNA origami purification

All purified DNA origamis were stored in the DNA origami storage buffer composed of 10 mM tris acetate, 10 mM magnesium acetate, 1 mM EDTA unless otherwise stated. All origamis were stored at 4°C and analysed within 2 weeks. All purification procedures were performed in 1.5 ml centrifuge tubes unless otherwise specified.

SPRI beads selection

Two brands of commercially available Solid-phase reversible immobilization (SPRI) beads were tested throughout this study: HighPrep™ PCR Clean-up System (AC-60050; Magbio) and SPRIselect (B23318; Beckman Coulter). The same procedure was used for both SPRI beads.

Prior to purification, the bead solution was allowed to warm up to room temperate on the bench at room temperature for 15 minutes. The bead solution was then vortexed for 30 seconds until a

homogenous colour was observed. The SPRI volume ratio used here means that for every 100 µl of DNA origami solution (after folding), adding 100 µl of suspended SPRI beads will give a ratio of 1.0X.

The 40 µl of folded DNA origami was mixed with 32 µl of the SPRI bead solution to achieve the 0.8X volume ratio with a final volume of 72 µl. The solution was mixed until a homogenous colour was achieved by flicking on the tube or pipette mixing, followed by 10 minutes incubation at room temperature on the bench. The tube was then placed on a magnetic separation rack (commercially available or homemade with 3D printer) for 5 minutes. The magnet pulled the SPRI beads from the solution to form a pellet at the corner of the tube. The clear supernatant was then extracted by pipette and discarded without disturbing the beads pellet. Then, two washes with 500 µl of 80% (v/v) ethanol were performed and all excess ethanol from the tube was then carefully removed with a pipette. The tube with the beads pellet was removed from the magnetic separation rack and sat on bench for 5 minutes to air dry. The dry pellet was then resuspended in 40 µl of the DNA origami storage buffer through pipette mixing to elute the DNA origami from the SPRI beads, followed by 5 minutes of incubation time. The tube was placed back on the magnetic separation rack and the magnet caused the beads to pellet at the corner of the centrifuge tube. A pipette was then used to aspirate the supernatant and transfer it to a PCR tube. The purified DNA origami was then thermally de-clumped, i.e. the PCR tube was heated to 50°C using a thermal cycler for 2 mins, followed by a gradual decrease of the temperature to 20°C at the rate of 3°C/min. The temperature was then further decreased down to 4°C until use. The purified DNA origami (40 µl in 1.5 ml tube) can also be de-clumped through vortex for 1 minute.

A homemade buffer containing 18% (w/v) PEG 8000, 1M NaCl, 10mM Tris-Cl, 1mM EDTA, pH 8.0 was used and exchanged with the HighPrep SPRI beads buffer for the purpose of condition screening in Figure S4. The buffer exchange was carried out by centrifugating 1ml of the HighPrep SPRI beads at 10,000g for 10 minutes, the buffer was then aspirated and replaced with 1ml of homemade buffer, followed by vortex to mix the beads with the replaced buffer.

S-400 HR spin column filtration

Microspin™ S-400 HR spin columns (27-5140-01; Cytiva) were used to purify the DNA origami. The spin column was set-up according to the manufacturer instructions. After the removal of the storage solution from the column by centrifugation at 750g for 1 minute, the spin column was equilibrated with DNA origami storage buffer by adding 200 µl to the column followed by centrifugation at 750g for 1 minute. The equilibration step was repeated 2 more times, and all

the eluates collected were discarded. The column was then transferred and inserted into a clean centrifuge tube, then 40 µl of the folded DNA origami was added to the column and spun down at 750g for 1 minute. The eluate from the column was collected which contained the purified DNA origami. The volume of the purified DNA origami was checked with a pipette, if the volume was less than 40 µl, additional DNA origami storage buffer was added to bring the volume to 40 µl.

Molecular weight cut-off (MWCO) membrane filtration

Two different 100 kDa MWCO filtration units were tested, the Amicon Ultra-0.5 Centrifugal Filter Unit (R0PB46526; Sigma-Aldrich) (referred to as MWCO-1) and the Vivaspin® 500 Centrifugal Concentrator (VS0142; Sartorius) (referred to as MWCO-2). The same purification procedure was used for both.

The column was set up as instructed by the manufacturer, then 400 µl of the DNA storage buffer was first added to the centrifugal cartridge followed by 40 µl of the folded DNA origami solution. The unit was then centrifuged at 15,000g for 10 minutes, the filtrate was discarded, and 400 µl of the DNA storage buffer was added to the centrifugal cartridge. This process was repeated 2 more times. Afterwards, the purified DNA origami solution was collected with a pipette. The volume of the purified DNA origami was checked with a pipette, and if the volume was less than 40 µl, additional DNA origami storage buffer was added to bring the volume to 40 µl.

PEG precipitation

For the purification of DNA origami by poly(ethylene) glycol (PEG) precipitation, the method described by Stahl *et al.* [6] was used without modifications. The 15% PEG precipitation buffer was prepared ahead of purification; it contained 15% (w/v) PEG 8000 (89510; Sigma-Aldrich), 5 mM Tris, 1 mM EDTA and 505 mM NaCl. The components were mixed and incubated at 85°C for 10 minutes and placed on tube roller for 2 hours.

The 40 µl folded DNA origami was further diluted down to 200 µl by adding 160 µl DNA origami storage buffer, then 600 µl of a buffer containing 5 mM Tris-HCl (pH 8.0), 1 mM EDTA, 20 mM $MgCl_2$, and 5 mM NaCl was added to the diluted folded DNA origami solution. Then 800 µl of the 15% PEG precipitation buffer was added to the diluted folded DNA origami solution, followed by mixing by flicking the tube. The mixture was centrifuged at 10,000g for 15 minutes, and the supernatant carefully removed without disturbing the pellet. The pellet was then resuspended in 800 µl of buffer containing 5 mM Tris-HCl (pH 8.0), 1 mM EDTA, 20 mM $MgCl_2$ and 5 mM NaCl, followed by the addition of 800 µl 15% PEG precipitation buffer, mixed and then centrifuged at 10,000g for 15 minutes. This step was repeated 2 more times. After the last

centrifugation step and the removal of the supernatant, the pellet was resuspended in 40 µl of DNA origami storage buffer.

Phase separation

For the purification of the DNA origami via phase separation method, the method described by Masukawa *et al.* [7] was used without modifications. Two buffers were required for this method. The first buffer was Dextran PEG buffer containing 8.3% (w/v) PEG 6000 (81255; Sigma-Aldrich), 0.83% (w/v) Dextran 200,000 (31398; Sigma-Aldrich), 40 mM tris acetate, 1 mM EDTA (15558042; Thermo fisher), and 5 mM magnesium acetate at pH 8.3. The second buffer was the PEG buffer containing 8.3% (w/v) PEG 6000, 40 mM tris acetate, 1 mM EDTA and 5 mM magnesium acetate at pH 8.3.

After the folding reaction, 320 µl of the Dextran PEG buffer was added to a 40 µl solution containing the folded DNA origami. The mixture was vortexed for 30 seconds before being centrifuged at 1700g for 1 minute. 240 µl of the supernatant was then removed and discarded, and 240 µl of PEG buffer was added to the tube. The tube was mixed again via vortex shaking for 30 seconds and centrifuged at 1700g for 1 minute. 200 µl of the supernatant was then removed and discarded, and 200 µl of PEG buffer added to the tube. The tube was vortexed again for 30 seconds and centrifuged at 1700g for 1 minute. 280 µl of the supernatant was removed and discarded. The remaining 40 µl solution was considered the purified DNA origami.

Ethanol precipitation

For the purification of the DNA origami via ethanol precipitation, the method described by Lei *et al.* [8] was used without modifications. For this method, the precipitation buffer contained 60% (v/v) ethanol, 10 mM Tris-HCl, 5 mM $MgCl_2$ and 1 mM EDTA at pH 8.0.

After the folding reaction, 40 µl of the ethanol precipitation buffer was added to the folded DNA origami solution, the tube was then centrifuged at 4500g for 30 minutes at room temperature. The supernatant was discarded and the pellet was resuspended in 40 µl of the DNA origami storage buffer.

Size Exclusion Chromatography (SEC)

A custom packed Sephacryl S-500 HR column was used. The column was packed according to the manufacturer instructions. The column bed volume was 40 ml, and the column inner diameter was 16 mm, the resin Sephacryl S-500 HR (17061310; Cytiva) was packed inside the column at a compression ratio of 1.15X. An AKTA pure system was used to facilitate and monitor the process. The column was equilibrated with the DNA origami storage buffer by running the column through 2 column volumes of DNA origami storage buffer. 320 µl of folded DNA origami solution was diluted to 1 ml in DNA origami storage buffer. The diluted folded DNA

origami solution was loaded onto the column through the AKTA, and the purification was carried out at flow rate of 0.1 ml/min Different fractions of eluate were collected based on the A_{280} trace. Fractions corresponding to the DNA origami were pooled and concentrated first via 3,000 MWCO Vivaspin® 6 Centrifugal Concentrator (VS0691; Sartorius) and then via the 3,000 MWCO Vivaspin® 500 Centrifugal Concentrator (VS0191; Sartorius) down to 40 µl.

Automated purification via liquid handling robot

For the automated purification using SPRI beads, the liquid handling system Biomek NX^P Automated Workstation (Beckman Coulter) was used. The DNA origami was folded inside a 96 well plate with each well containing 20 µl of folded DNA origami mixture. The entire purification procedure including magnet beads separation, ethanol rinse, incubation and liquid transfer was set-up in the liquid handling system. Briefly, upon setting up the automation protocol, the folded DNA origami PCR plate was placed in a specific spot, the liquid handling robot first resuspend the SPRI beads 3 times and added 16 µl of SPRI beads (SPRI volume ratio at 0.8X) to the PCR plates and resuspended the mixture. After incubation, the plate was transferred by the robot to a magnetic plate, the supernatant was aspirated and discarded, followed by rinsing steps, due to the volume limit of a 96 well plate, only 120 µl of 80% ethanol was used to rinse the beads pellet. The plates were moved by the robot away from the magnetic plate and 20 µl of DNA origami storage buffer was added, mixed and the plate was transferred back to the magnetic plate. The supernatant containing the purified DNA origami was aspirated and stored in a clean PCR plate by the robot. The final beads elution volume was 20 µl to maintain the input and output volume consistency. The eluted plate from the liquid handling robot was then subjected to thermal de-clumping procedure as described above.

DNA origami yield measurement

Absorption spectroscopy

The concentration of the purified DNA origami was measured via absorption spectroscopy using a NanoDrop™ 2000c Spectrophotometer (Thermo Fisher). The instrument was blanked with the DNA origami storage buffer. 2 µl of the sample was dotted onto the measurement platform, and the measurement was performed using an extinction coefficient of 33 mg/ml for $A_{260} = 1$.

Fluorescence

The concentration of the DNA origami was also measured with a fluorescence-based method which allows the measurement of numerous samples in a 96 wells format. The QuantiFluor® ONE dsDNA System (E4871; Promega) was used to measure the concentration of the purified DNA origami after the automated purification step via pipette robot. The set-up and procedure

were followed as instructed by the manufacturer, but with multiple changes to adapt for the DNA origami: 1. The 1X TE buffer was replaced with the DNA origami storage buffer; 2. Instead of the 48.5 kbp λ dsDNA provided by the kit to use as the calibration curve, a known mass (72, 36 and 18 ng) of the purified DNA origami (from S-400 HR spin column purification) was used.

The measurement was carried out in the Spark® multimode microplate reader (TECAN) with excitation at 360 nm and emission at 535 nm. The plate was shaken for 10 seconds in double orbital mode prior to reading, then each well was read 16 times at different position and the fluorescent average was calculated from the 16 readings.

Size selection of the DNA ladder

The GeneRuler 1 kb Plus DNA Ladder (SM1331; Thermo Fisher) was used with the HighPrep™ PCR Clean-up System to demonstrate the size selective behaviour of the SPRI beads. Briefly, three ratios were selected (0.4X, 1.0X and 4.0X), a total of 1000 ng of the DNA ladder was dilute in 20 µl volume. The procedure of the SPRI beads selection follows the same procedure as above in DNA origami purification, except the thermal de-clump step was skipped.

Agarose Gel analysis

For the agarose gel analysis of the DNA origami, a 0.8% agarose gel was used. The high purity agarose (16500500; Thermo Fisher) was mixed with 0.5X TBE, 10 mM $MgCl_2$ buffer in an Erlenmeyer flask. The agarose was melted in microwave oven at full power for 45 seconds, and immediately poured into the casting module of the agarose gel electrophoresis system (Mini-Sub Cell GT Systems; Bio-Rad). Either a fixed mass (25 ng) of the purified DNA origami or a fixed volume (4 µl) of the purified DNA origami was used. The purified DNA origami was mixed with 2 µl of the loading dye (B7025S; NEB) and the volume was brought up to a total of 8 µl with DNA origami storage buffer. The M13mp18 ssDNA scaffold was prepared in the same way with a fixed mass of 25 ng for gel analysis. The gel was submerged in the 0.5X TBE, 10 mM $MgCl_2$ buffer inside a cold room (4°C), the samples were loaded with pipette, and 70 V was applied for 90 minutes. The gel was then transferred to a foil-covered container containing 30 ml of 1X TAE buffer, and 10 µl of the Diamond nucleic acid dye (H1181; Promega) was used to stain the gel for at least 30 minutes.

For the gel analysis of the DNA ladder, a 0.8% agarose gel was used. The agarose was mixed with 1X TAE, melted in a microwave oven at full power for 45 seconds, and casted with the gel casting module as described above. The samples were prepared similarly to DNA origami but with a different loading dye (B7024S; NEB). The gel was ran at 60 V for 60 minutes, followed by gel staining with Diamond nucleic acid dye as described above.

The gel imaging was carried out with the InGenius LHR Gel Doc System (Syngene).

Further analysis of the gel such as densitometry analysis was done via ImageJ.

Origami-protein mixture clean-up

DNA origami purification from excess functional molecules: folded DNA origami structures were purified using SPRI beads as described. The concentration of origami solution was checked with absorption spectroscopy. 24 nM of C-reactive protein (CRP; 140-11R; Lee-Bio) was added to 12 nM of purified 4FST origami. The clean-up of the mixture follows the procedure described above.

SDS-PAGE analysis

A gradient sodium dodecyl sulphate polyacrylamide gel electrophoresis (SDS-PAGE) gel was prepared by first preparing a 4% and 20% polyacrylamide gel by mixing 1 M Tris-base, 0.3% SDS (pH adjusted with HCl to 8.45) with 30% acrylamide/bis-acrylamide (29:1), 1% ammonium persulfate and 0.1% Tetramethylethylenediamine (TEMED).

For the preparation of the gradient gel, 1 volume portion of the 4% gel mixture was aspirated with a 10 ml serological pipette with an electronic pipette controller, then the same pipette was used to aspirate 1 volume portion of the 20% gel mixture. The serological pipette was withdrawn from the gel buffer tube, an air bubble was then generated by gently pressing the aspiration button from the controller, and the air bubble was allowed to raise. After the air bubble disappeared, the gradient gel mixture was dispensed to the narrow gap in the sandwiched 1.0 mm glass plates. A 1 mm size comb was inserted, and the gel was allowed to solidify. 15 µl of samples were mixed with 6 µl of 3X sample loading dye (187.5 mM Tris-HCl, 6% SDS, 30% glycerol, pH 6.8) so that the final mixture contains a 1X loading dye buffer and 12 ng of materials. 1X cathode (0.1M Tris base, 0.1M Tricine, 0.1% SDS, pH 8.3; J60992.K2; Alfa Aesar) and 1X anode buffer (0.2M Tris-HCl, pH 8.9) were used for running the gel. 20 µl of sample was loaded into the wells after the gel was solidified. The gel was then run at 200 V for 30 minutes. Staining of the gel was done using a silver staining kit (24641; Thermo scientific) or InstantBlue® Coomassie Protein Stain (ab119211; abcam) following the manufacturer's instruction. The silver stained all organic components including proteins and DNA. The stained gel was visualised and imaged using an InGenius LHR Gel Doc System (Syngene).

Atomic Force Microscopy (AFM)

Freshly cleaved mica discs were pre-treated with 5 µl of 10 mM $NiCl_2$, then immediately 5 µl of the DNA origami samples were deposited onto the mica by pipetting directly into the $NiCl_2$

solution and left on bench for 5 mins. The samples were topped up with 100 µl of DNA origami storage buffer. The DNA origami samples were imaged using a Bruker Dimension Fastscan Bio (Santa Barbara, CA, USA) with Fastscan-D-ss or ScanAsyst-Fluid+ cantilevers containing a Si tip. The imaging was carried out via PeakForce tapping with ScanAsyst™ liquid imaging mode via the Nanoscope software. All images acquired have a pixel resolution of 1024 x 1024, and the images were analysed with Nanoscope analysis 1.9.

Intact origami percentage calculation

To calculate the percentage of the intact 4FST origamis from AFM images, the following rules were used. See supporting information 2 for all the AFM images we used for this calculation.

- Only structures fully visible in the AFM images are considered.
- To be considered as an intact origami:
    - the 4FST is composed of 4 individual triangles coming together to form a square shape, and all 4 triangles must be intact.
    - The tile is symmetric.
    - The tile has all four edges, and they are all 80 nm.
- Everything else is considered as damaged.

Based on these rules, the table below summarises the number of origamis counted for each method, and the number of intact origamis.

Supporting Table S1. Quantification of origami structures from AFM images for each method.

Method Name	*Intact origami*	*Damaged origami*	*Total Origami Counted*
0.8X SPRI	133	13	146
S-400 HR	54	8	62
MWCO-1	54	55	108
MWCO-2	24	1	25
PEG precipitation	38	1	39
Ethanol Precipitation	59	8	67
Phase Separation	21	2	23
SEC	79	14	93

Streptavidin functionalised origami percentage calculation

To calculate the percentage of the streptavidin functionalised 4FST origami from AFM images, the following rules were used.

- Only structures fully visible in the AFM images are considered.
- To be considered as a single streptavidin-functionalised 4FST origami, it must be intact (according to the rules in Table S1).
- In cases where two 4FST origamis formed a dimer as a result of a single streptavidin bound to two biotinylated oligos of two separate 4FST origamis, it is counted as one.

Based on these rules, the table below summarises the number of origamis counted for the two methods.

Supporting Table S2. The quantification of streptavidin functionalised origami from AFM images for each method.

Method Name	Streptavidin functionalised origami	Total Origami Counted
0.8X SPRI	102	120
S-400 HR	49	105

CRP bound percentage of the CRP affimer functionalised origami calculation

To calculate the CRP bound percentage of the CRP-affimer functionalised 4FSF origami from AFM images, the following rules were used.

- Only structures fully visible in the AFM images are considered.
- To be considered as a CRP bound CRP affimer functionalised 4FSF origami complex, it must be intact (according to the rules in Table S1).

Based on these rules, the table below summarises the number of origamis counted for the two methods.

Supporting Table S3. The quantification of CRP bound CRP-affimer functionalised 4FSF origami from AFM images.

	Origami with CRP	Origami without CRP	Total Intact Origami Counted
4FSF origamis	45	52	97

Section 2: Supporting Figures

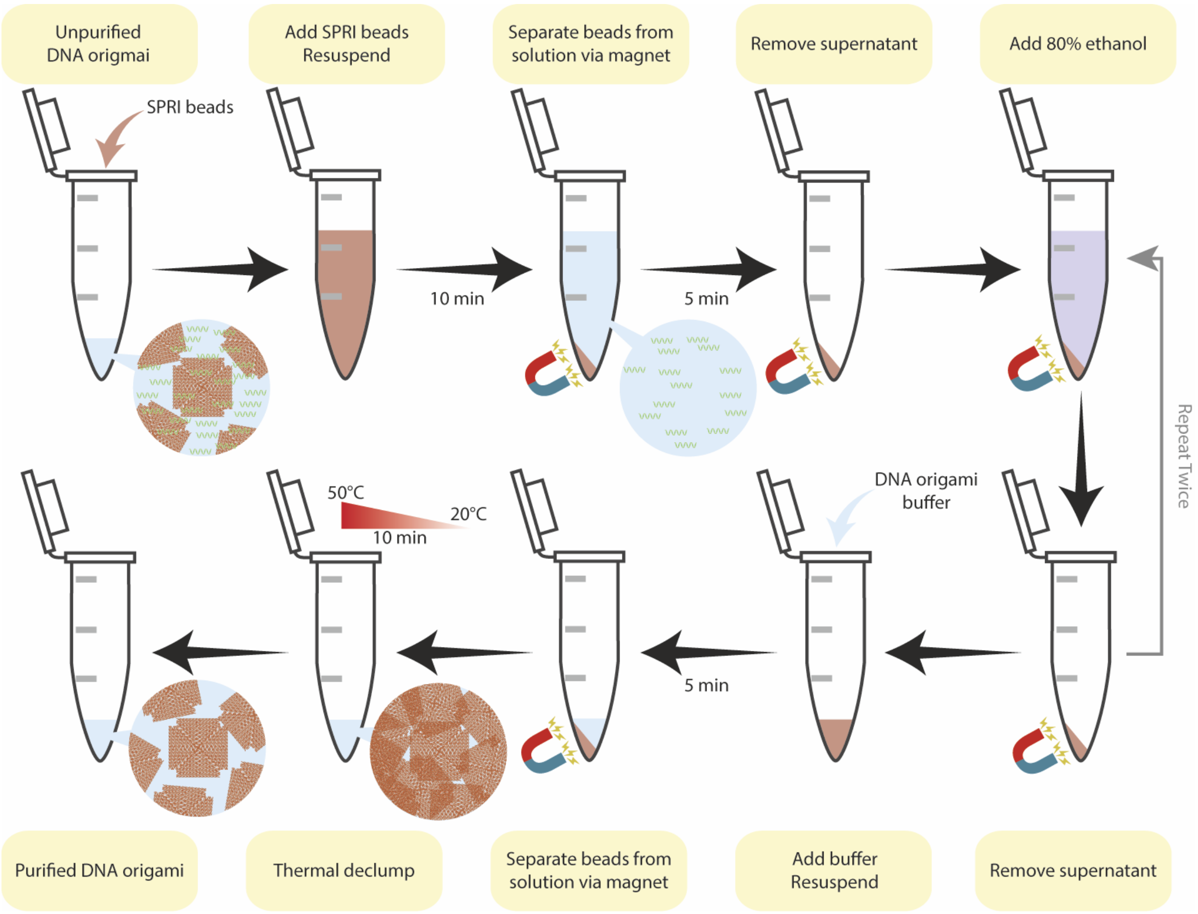

Supporting Figure S1. Schematic illustration of the SPRI DNA origami purification. The folded DNA origami is mixed with SPRI bead solution at a specific volume ratio. The SPRI beads are concentrated in one part of the tube by a magnet, and the DNA origami together with bead form a pellet with the aid of the magnet, but the excess staples stay in the supernatant and are removed from the mixture. The beads pellet is washed with 80% ethanol twice to remove excess salt contamination from the SPRI bead buffer and the folding reaction buffer. The purified DNA origami can then be eluted in a buffer of choice (here: 10 mM tris acetate, 10 mM magnesium acetate, 1 mM EDTA), and the eluted DNA origami is subject to thermal a de-clumping procedure to yield the final purified product.

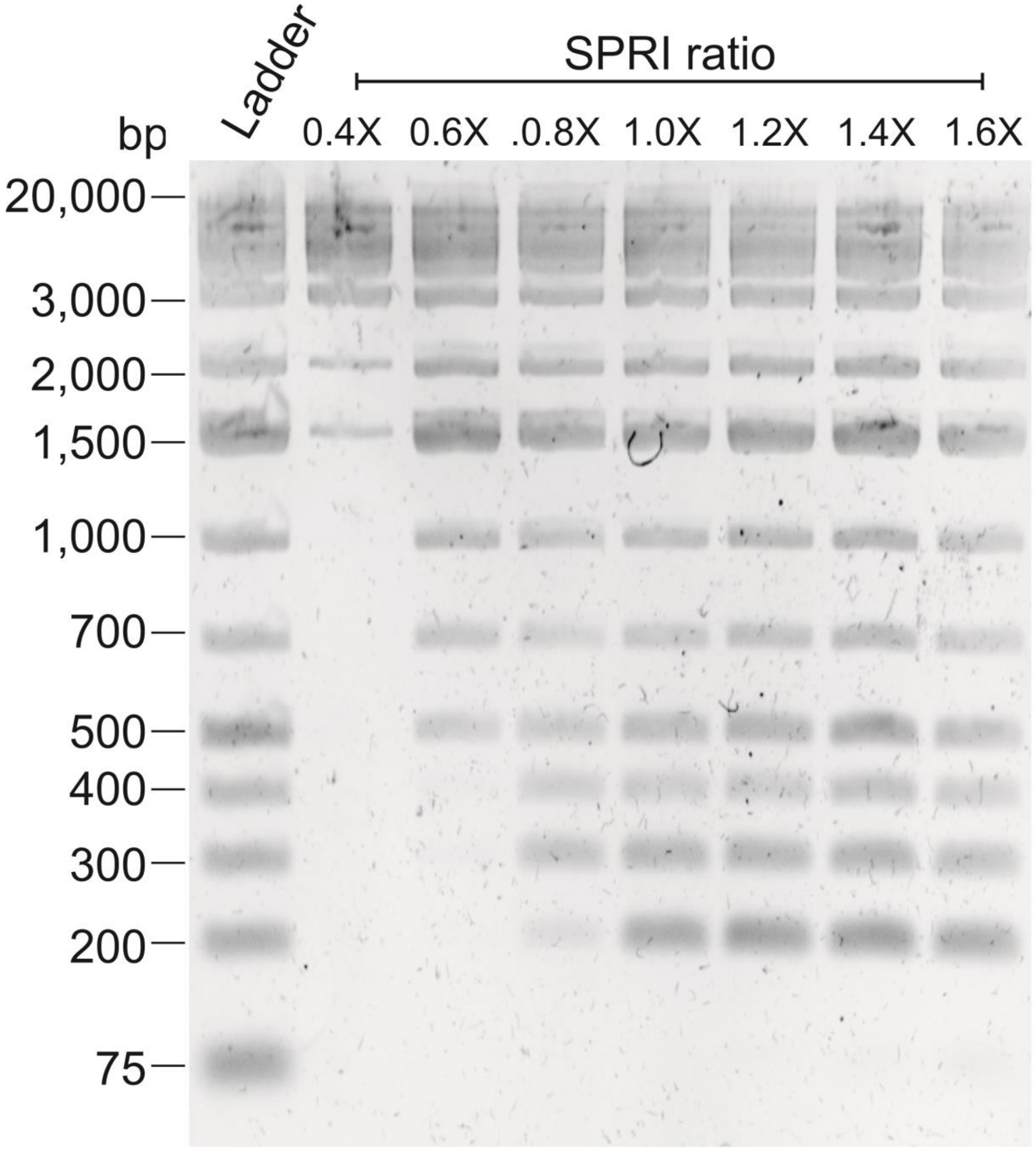

Supporting Figure S2. DNA size selection ability of the SPRI beads. The DNA ladder used here was mixed with different ratios of SPRI beads (0.4X to 1.6X). At SPRI ratio of 0.4X, the SPRI beads removed dsDNA smaller than 1,500 bp. Increasing the SPRI ratio to 0.6X and 0.8X caused it to remove dsDNA below 500 bp and 200 bp respectively. Further increasing the ratio (1.0X to 1.6X) removed dsDNA below 200 bp.

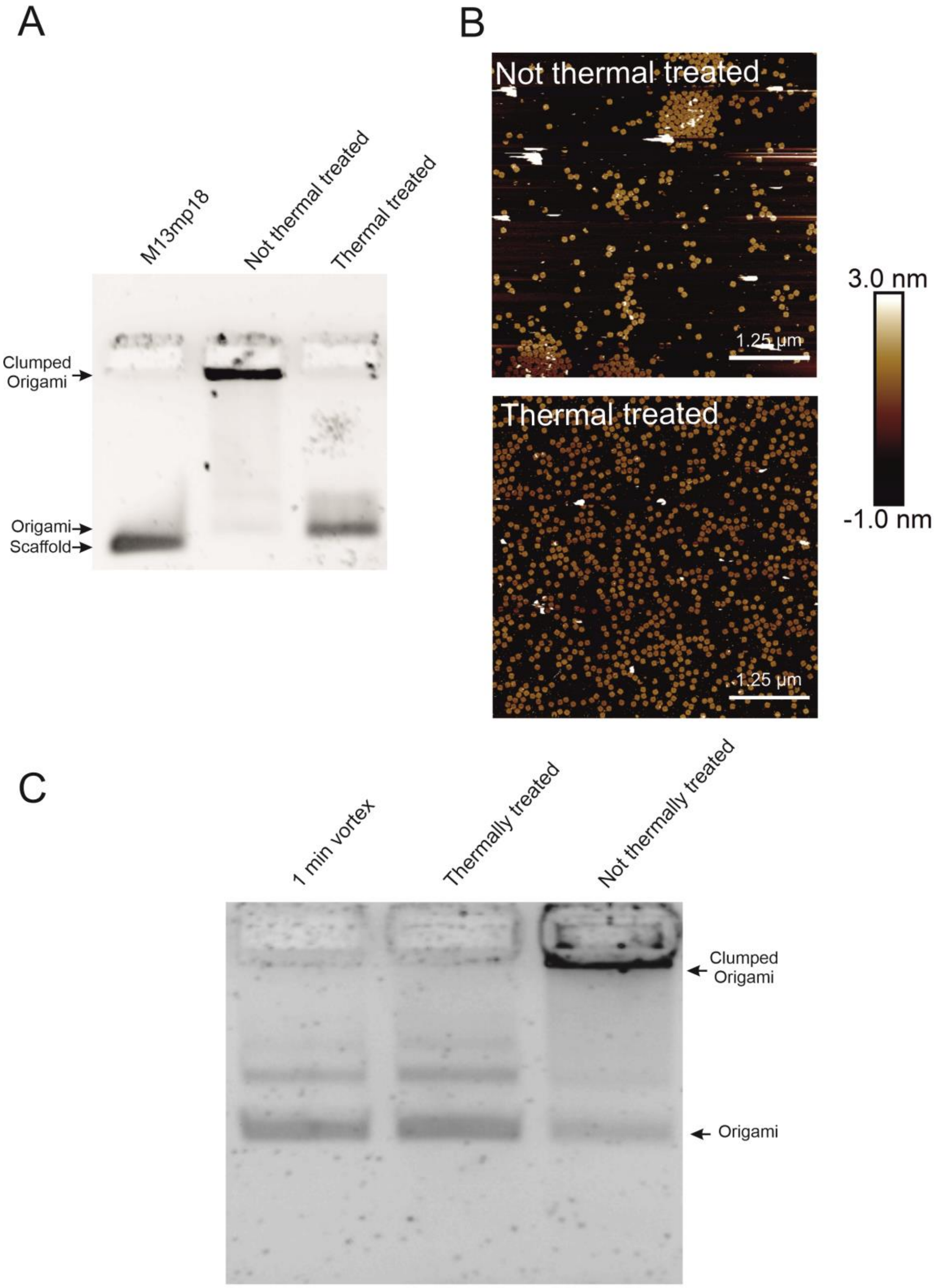

Supporting Figure S3. Thermal de-clumping of the SPRI-bead-purified DNA origami. (A) Agarose gel showing that without the thermal de-clumping treatment step, the 4FST DNA origamis were not able to migrate through the agarose gel, suggesting significant aggregation. In contrast, the thermal de-clumping treatment of the same sample enables the 4FST DNA origami to enter the agarose gel and migrate to the expected position. (B) AFM images of the thermal and not thermal treated 4FST DNA origami. The origami clumped together on the mica for the not thermally treated sample, whereas the thermally treated origami dispersed evenly across the mica surface. (C) The alternative way to de-clump the origamis is to vortex the 1.5 ml tube with 40 µl of the DNA origami solution for 1 min.

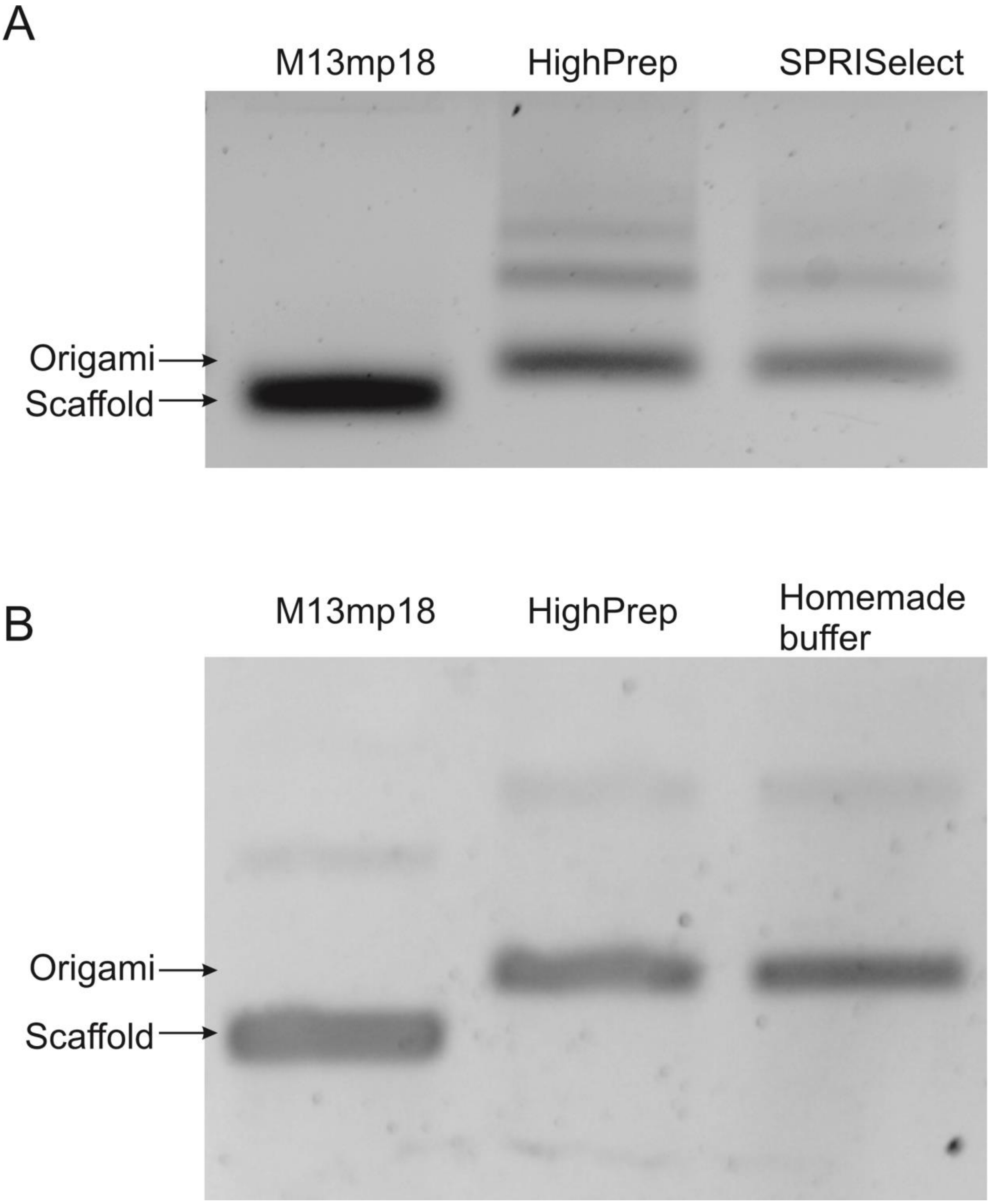

Supporting Figure S4. SPRI beads suppliers and conditions testing. (A) Two different suppliers of SPRI beads were tested (HighPrep and SPRISelect). Based on gel analysis, no differences in performance for the purification of the 4FST DNA origami were observed. (B) The HighPrep SPRI beads buffer was exchanged to a homemade buffer containing 18% (w/v) PEG 8000, 1M NaCl, 10mM Tris-Cl, 1mM EDTA, pH 8.0. The buffer exchanged SPRI beads was used to perform the purification of the DNA origami. HighPrep SPRI beads were used throughout the study unless stated otherwise.

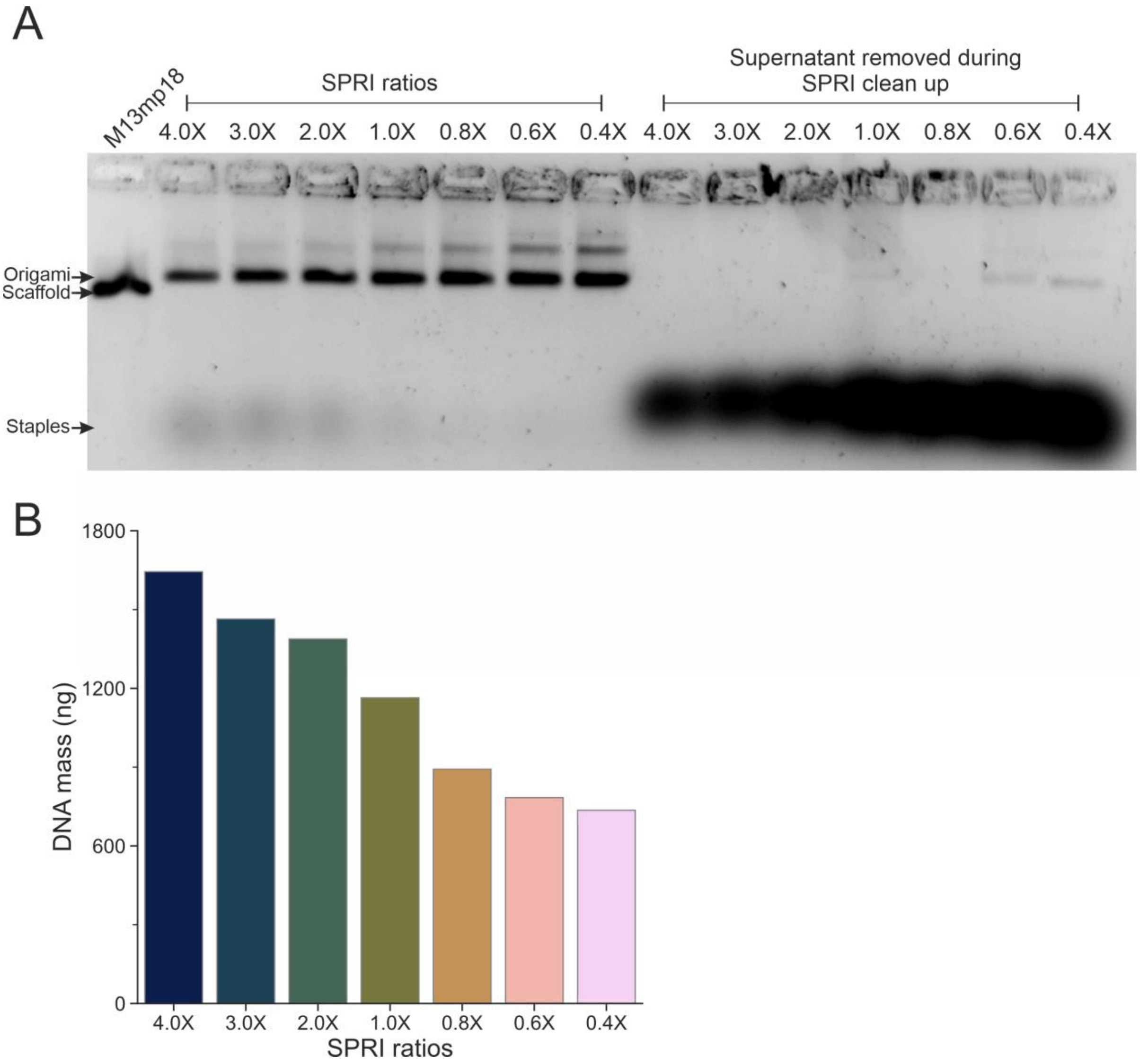

Supporting Figure S5. Volume ratio screening of the SPRI beads for the purification of the 4FST DNA origami. (A) Agarose gel image of Figure 2, but also including the supernatant of the purification. The excess staples from the folding reaction can be seen in the supernatant, demonstrating that the SPRI beads efficiently retained the DNA origami but not the staples. For the analysis of the supernatant, a fixed volume of sample was added instead of fixed DNA mass. This results in more staples and thus brighter bands for the lower volume ratios as the concentration of origami assembly sample is more diluted when the SPRI beads are added (*e.g.* the origami folding sample (40 µl) is diluted to 200 µl for a 4.0X ration versus to 56 µl for 0.4X). (B) The yield of the purified DNA origami measured by A_{260} absorbance. We note that although 4.0X had the highest yield, it could be seen from the gel that it was contaminated by the excess staples in the solution.

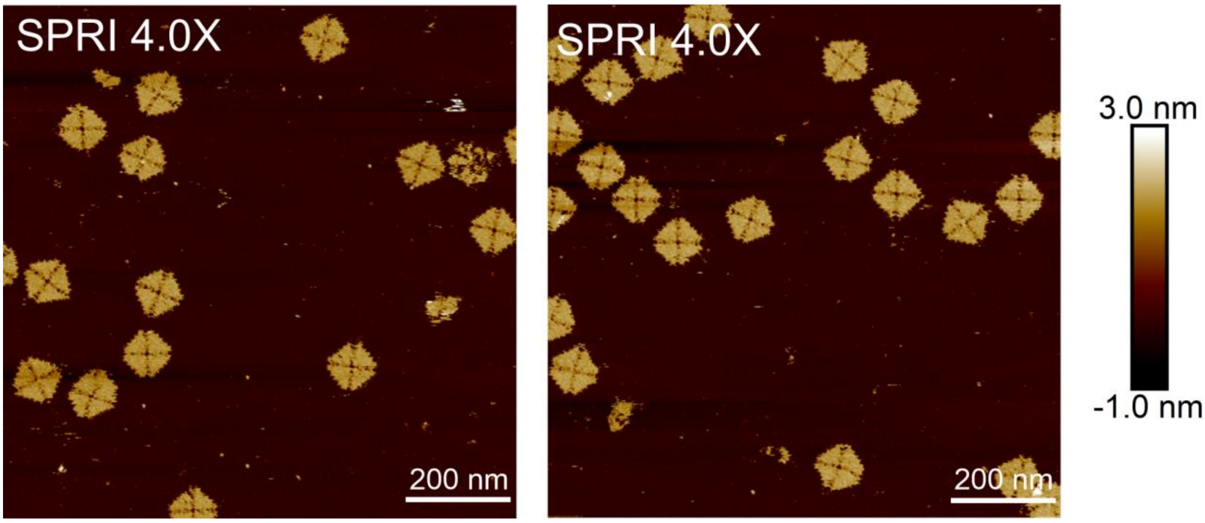

Supporting Figure S6. AFM images of 4FST DNA origami purified with 4.0X SPRI bead volume ratio.

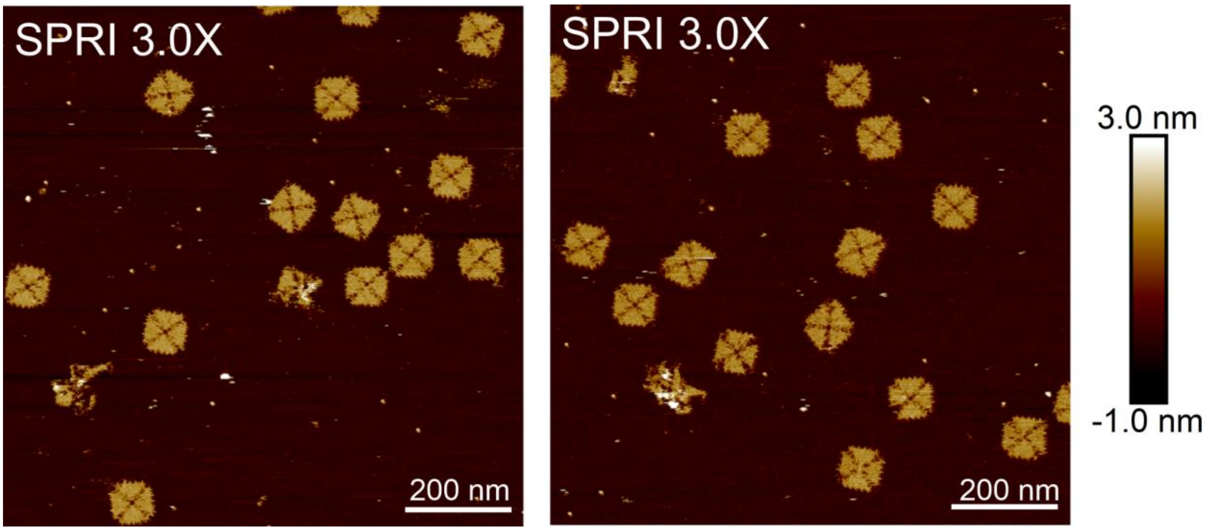

Supporting Figure S7. AFM images of 4FST DNA origami purified with 3.0X SPRI bead volume ratio.

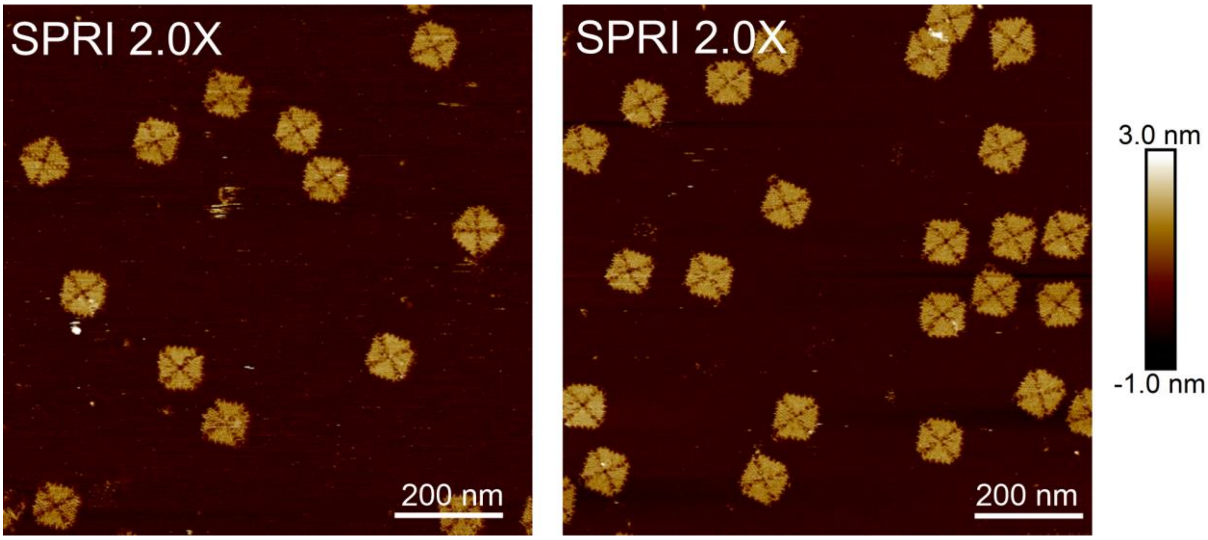

Supporting Figure S8. AFM images of 4FST DNA origami purified with 2.0X SPRI bead volume ratio.

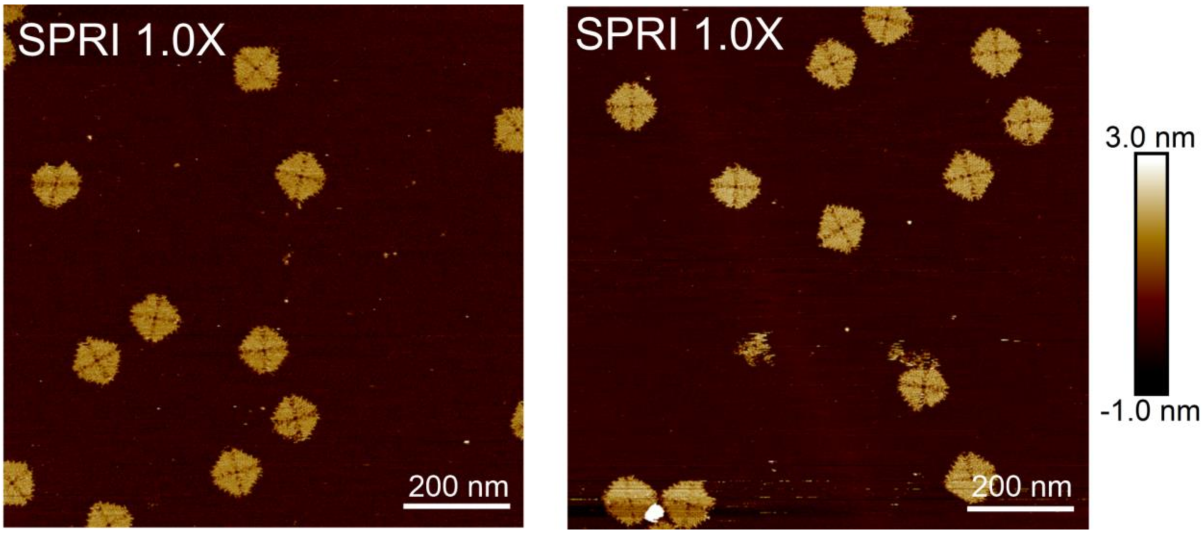

Supporting Figure S9. AFM images of 4FST DNA origami purified with 1.0X SPRI bead volume ratio.

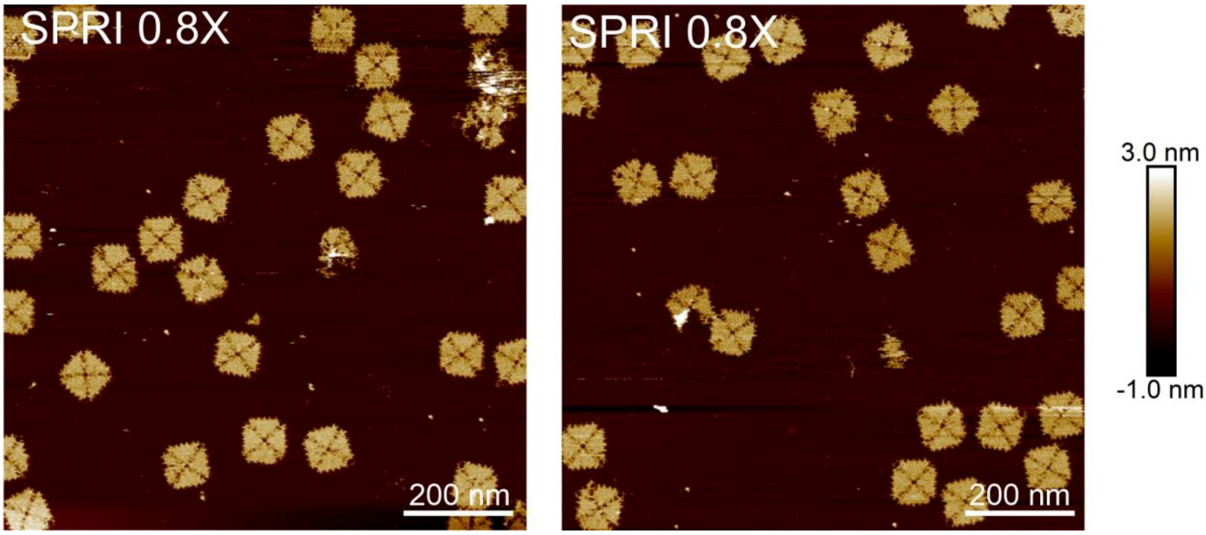

Supporting Figure S10. AFM images of 4FST DNA origami purified with 0.8X SPRI bead volume ratio.

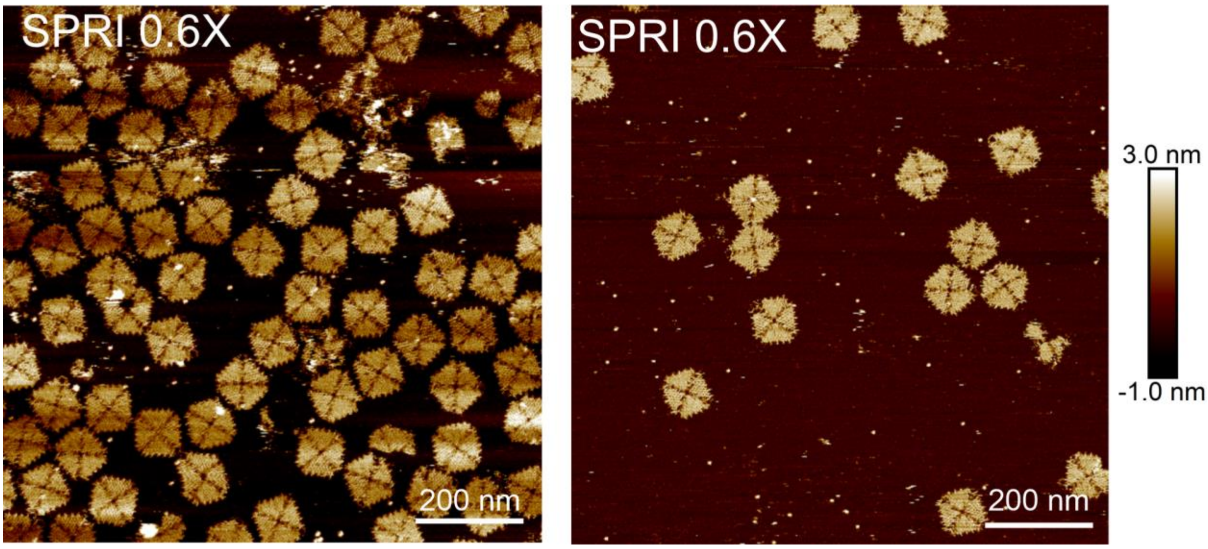

Supporting Figure S11. AFM images of 4FST DNA origami purified with 0.6X SPRI bead volume ratio.

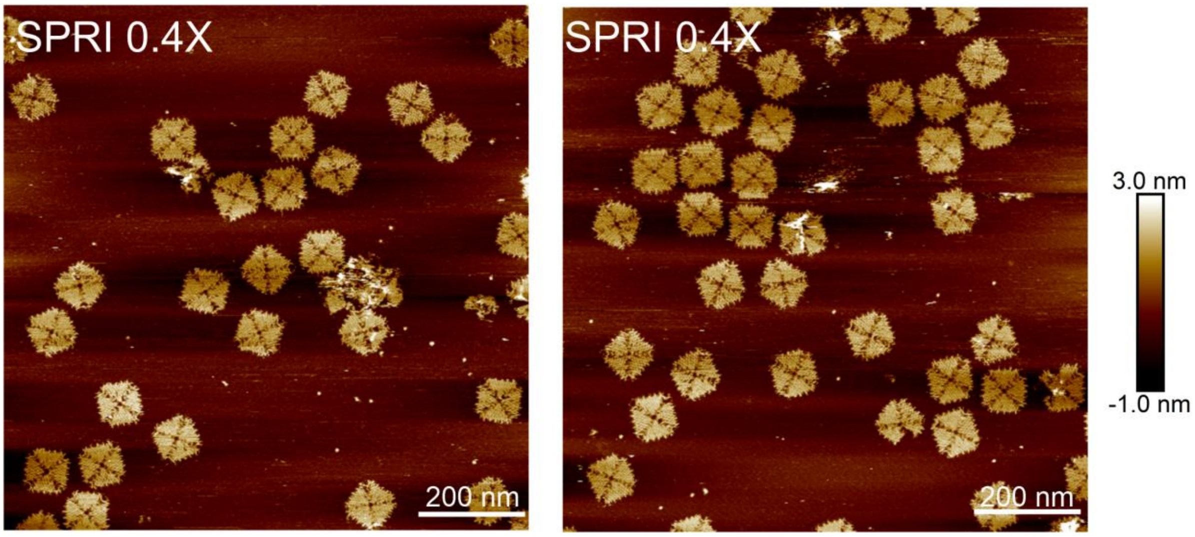

Supporting Figure S12. AFM images of 4FST DNA origami purified with 0.4X SPRI bead volume ratio.

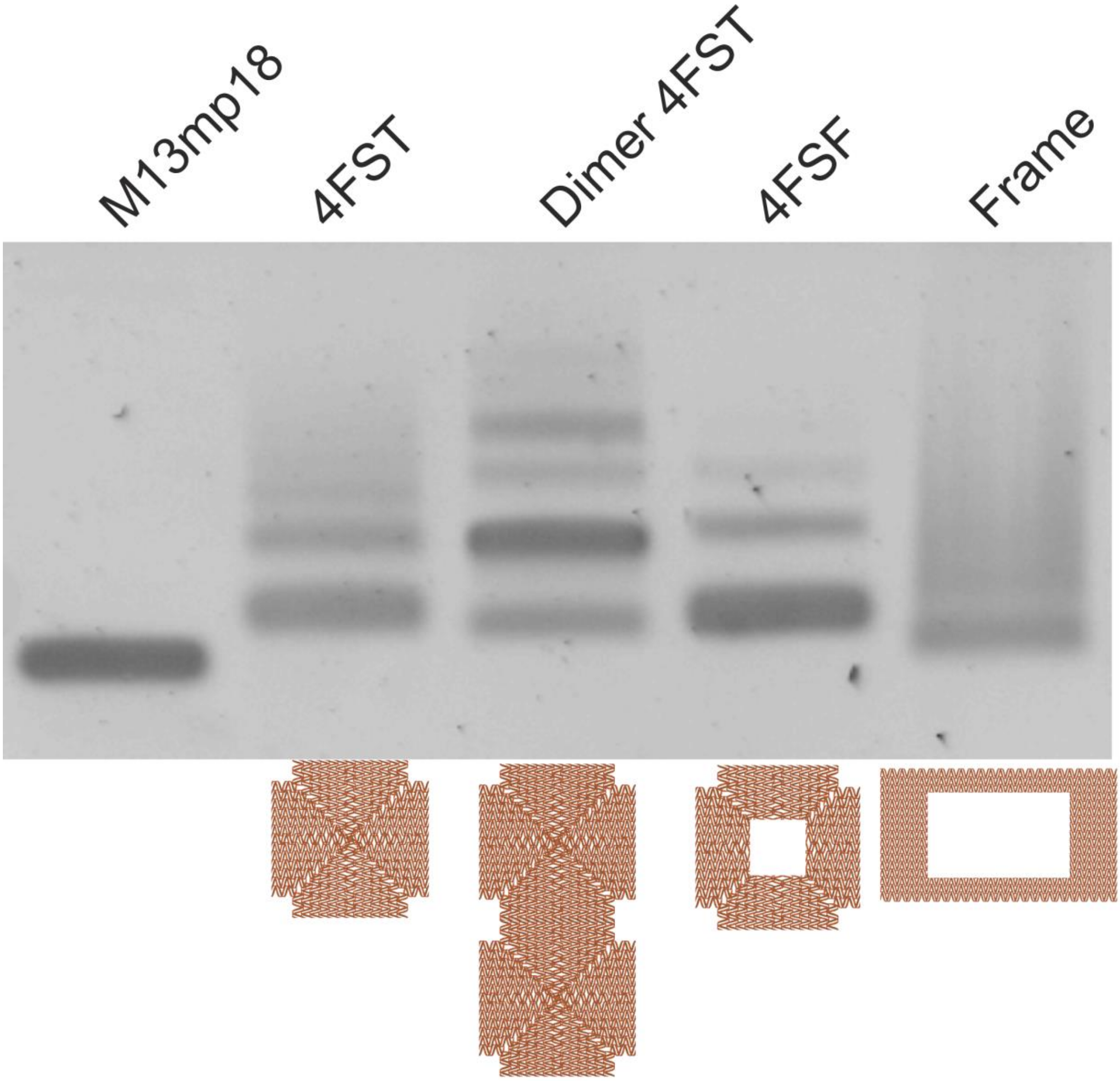

Supporting Figure S13. Agarose gel electrophoresis analysis of the SPRI-bead-purified DNA origami structures. All origami structures were purified with SPRI beads at a 0.8X volume ratio. The 4FST can dimerise to become the dimer 4FST. The 4FSF and frame origami both have a central cavity but of different dimensions. All structure migrate at the expected rate on the agarose gel.

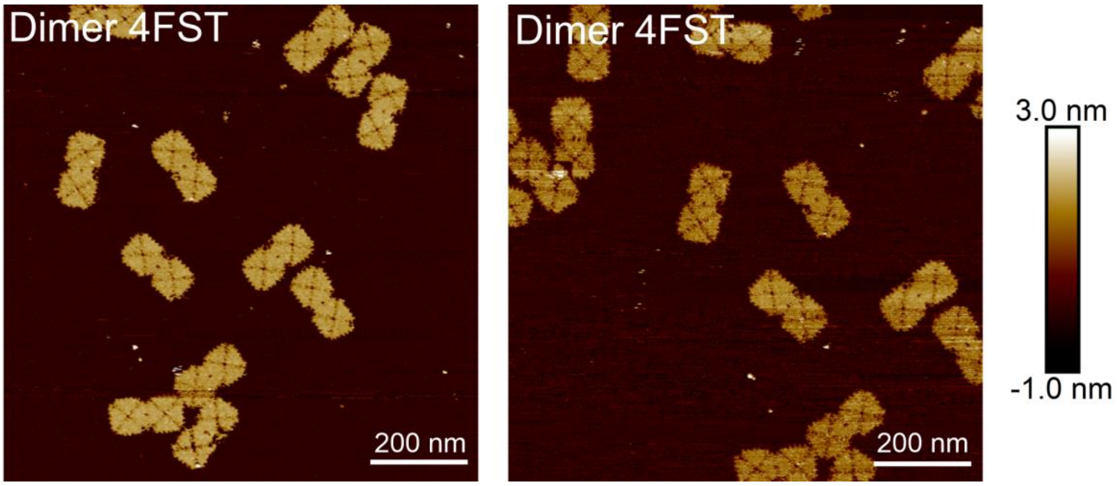

Supporting Figure S14. AFM images of the dimer 4FST DNA origami purified with 0.8X SPRI bead volume ratio.

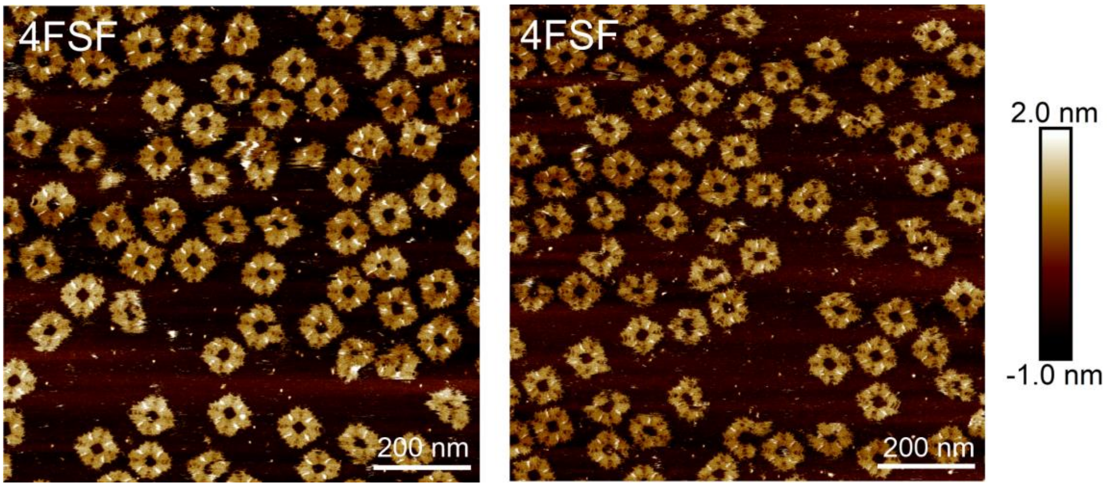

Supporting Figure S15. AFM images of the 4FSF DNA origami purified with 0.8X SPRI bead volume ratio.

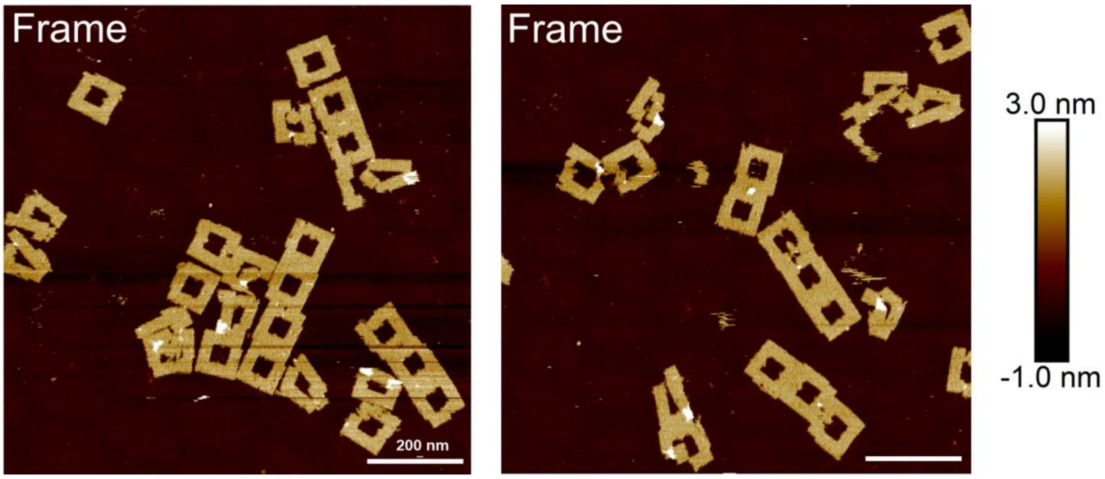

Supporting Figure S16. AFM images of the frame DNA origami purified with 0.8X SPRI bead volume ratio. Due to the structural issue of the DNA frame, it is easily collapsed can dimerise through stacking interaction. We have observed similar issues in our previous publications using the S-400 HR spin column filtration method to purify the origami [3, 5, 9].

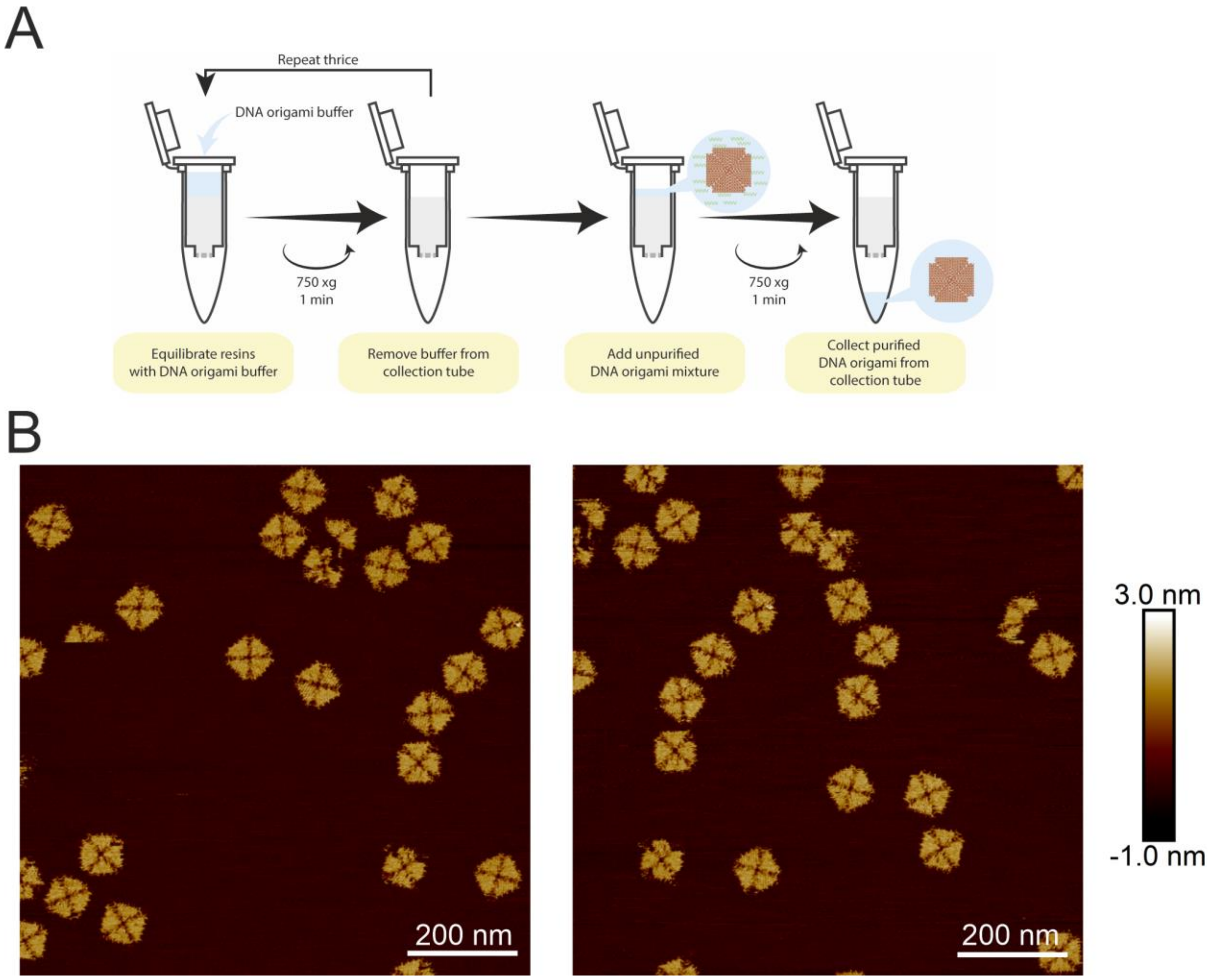

Supporting Figure S17. The purification of the 4FST DNA origami with S-400 HR spin column elution method. (A) Schematic illustration of the procedure. (B) AFM images of the S-400 HR spin column elution purified 4FST DNA origami.

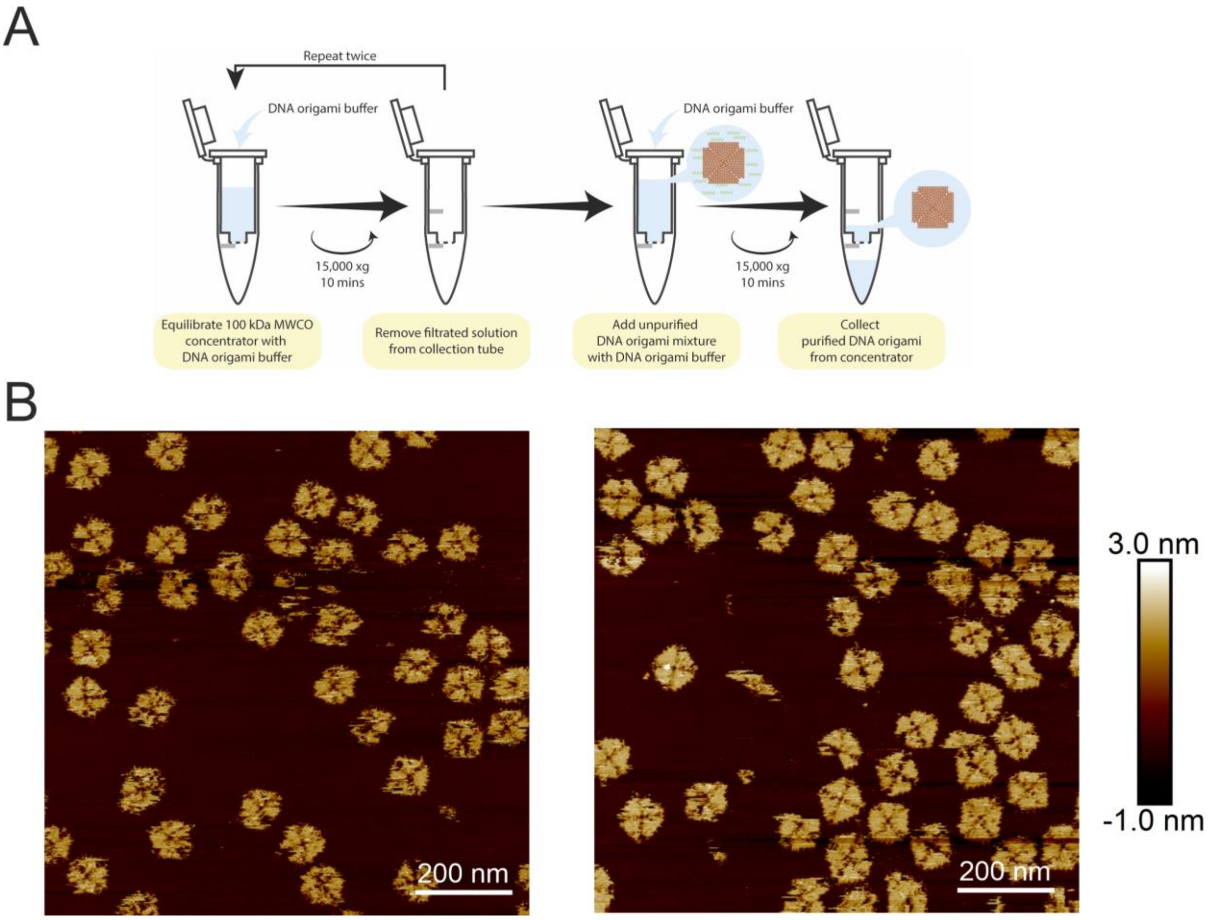

Supporting Figure S18. Purification of the 4FST DNA origami with the 100 kDa MWCO membrane filtration (Amicon Ultra-0.5 Centrifugal Filter Unit, MWCO-1) method. (A) Schematic illustration of the procedure. (B) AFM images of the 100 kDa MWCO purified 4FST DNA origami.

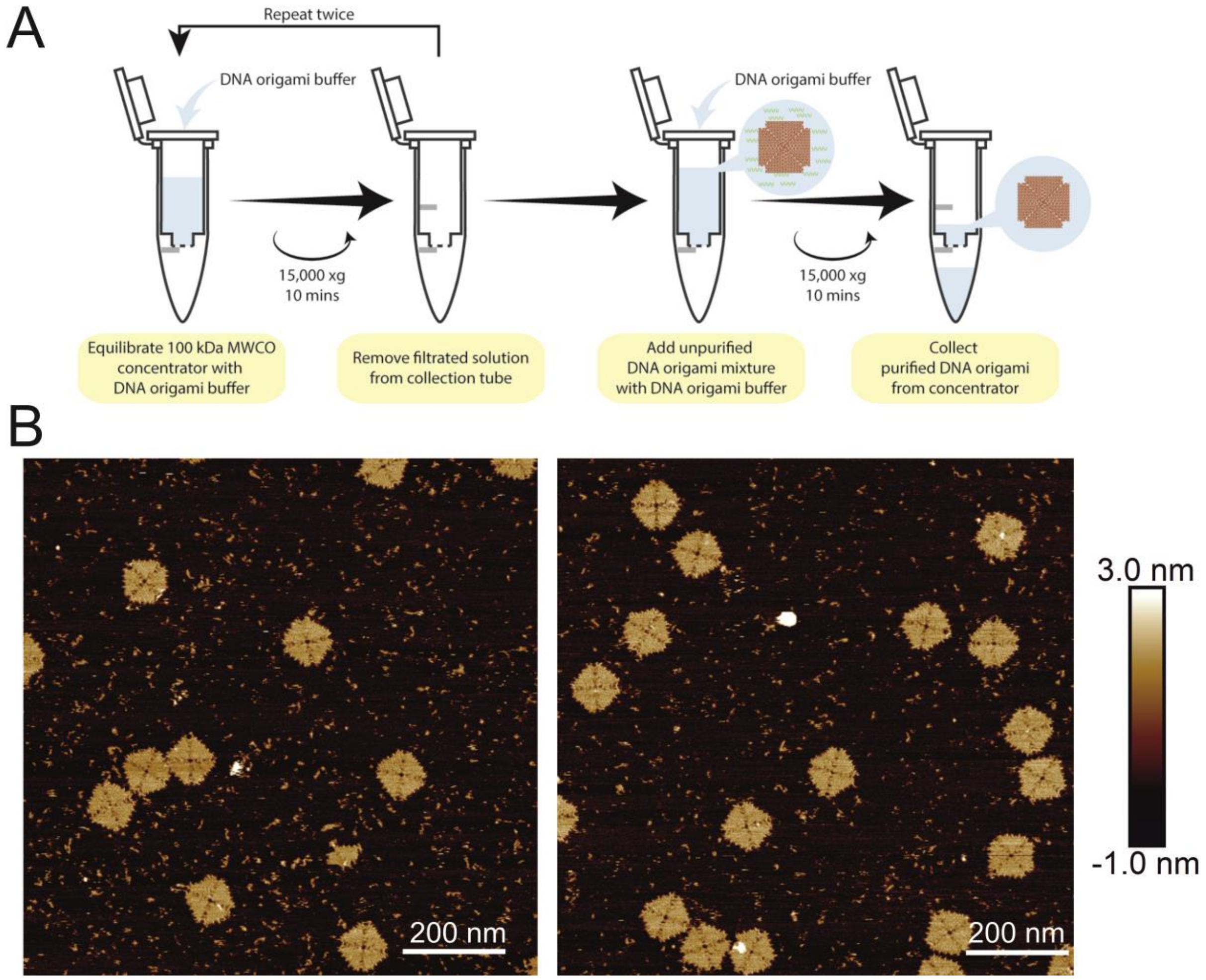

Supporting Figure S19. Purification of the 4FST DNA origami with the 100 kDa MWCO membrane filtration (Vivaspin® 500 Centrifugal Concentrator, MWCO-2) method. (A) Schematic illustration of the procedure. (B) AFM images of the 100 kDa MWCO purified 4FST DNA origami.

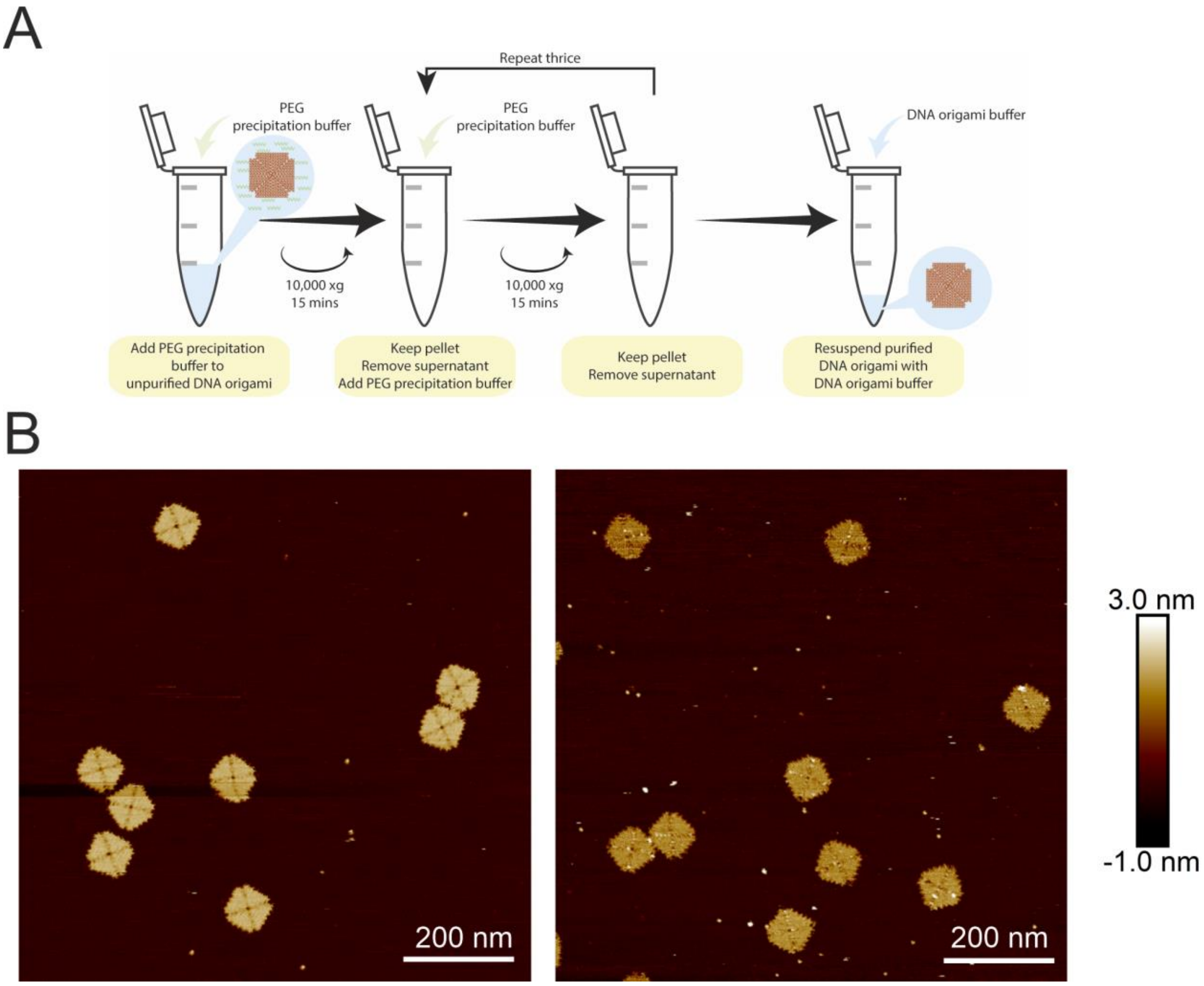

Supporting Figure S20. Purification of the 4FST DNA origami with the PEG precipitation method. (A) Schematic illustration of the procedure. (B) AFM images of the PEG precipitated 4FST DNA origami.

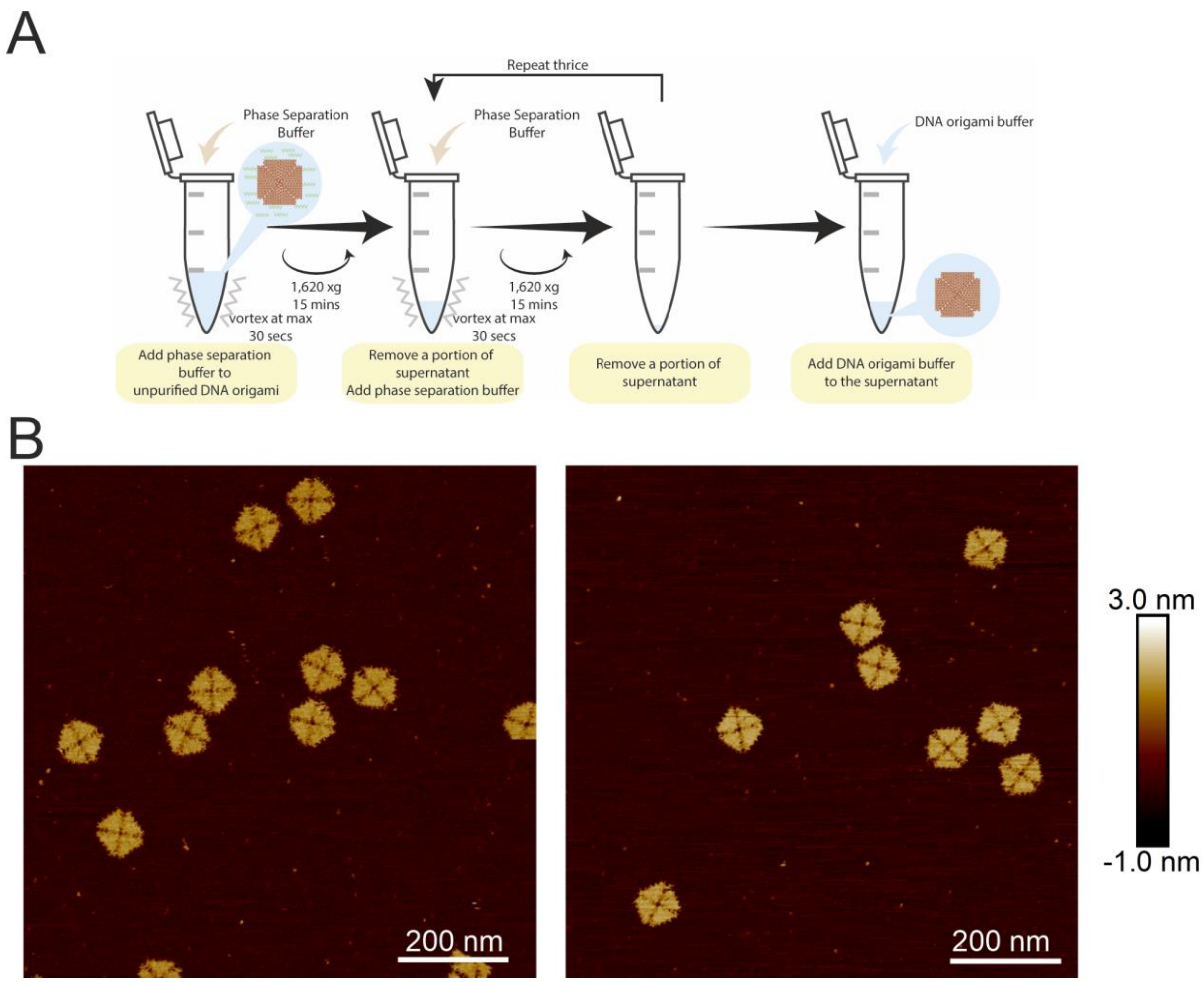

Supporting Figure S21. Purification of the 4FST DNA origami with the phase separation method. (A) Schematic illustration of the procedure. (B) AFM images of the phase separated 4FST DNA origami.

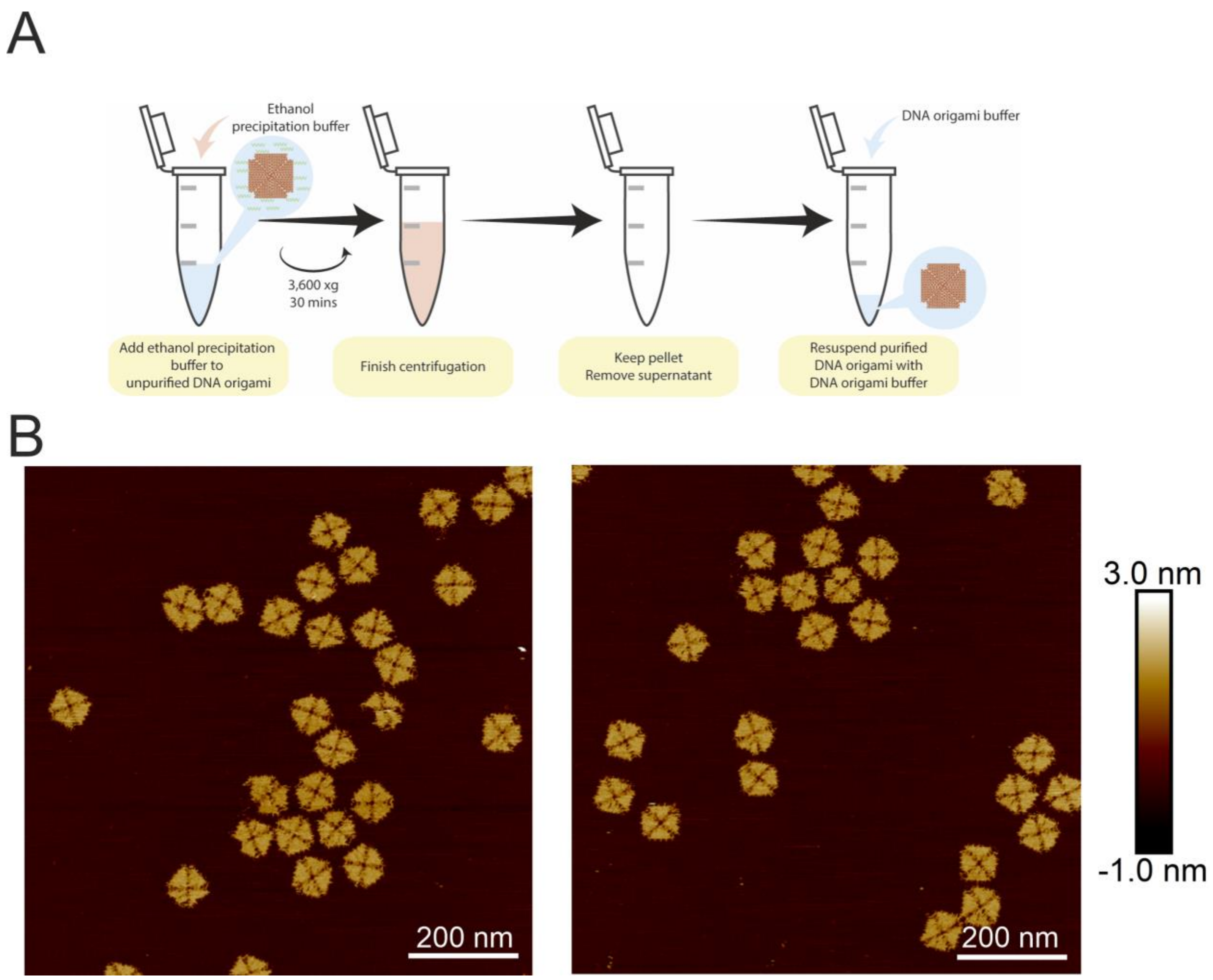

Supporting Figure S22. Purification of the 4FST DNA origami with the ethanol precipitation method. (A) Schematic illustration of the procedure. (B) AFM images of the ethanol precipitated 4FST DNA origami.

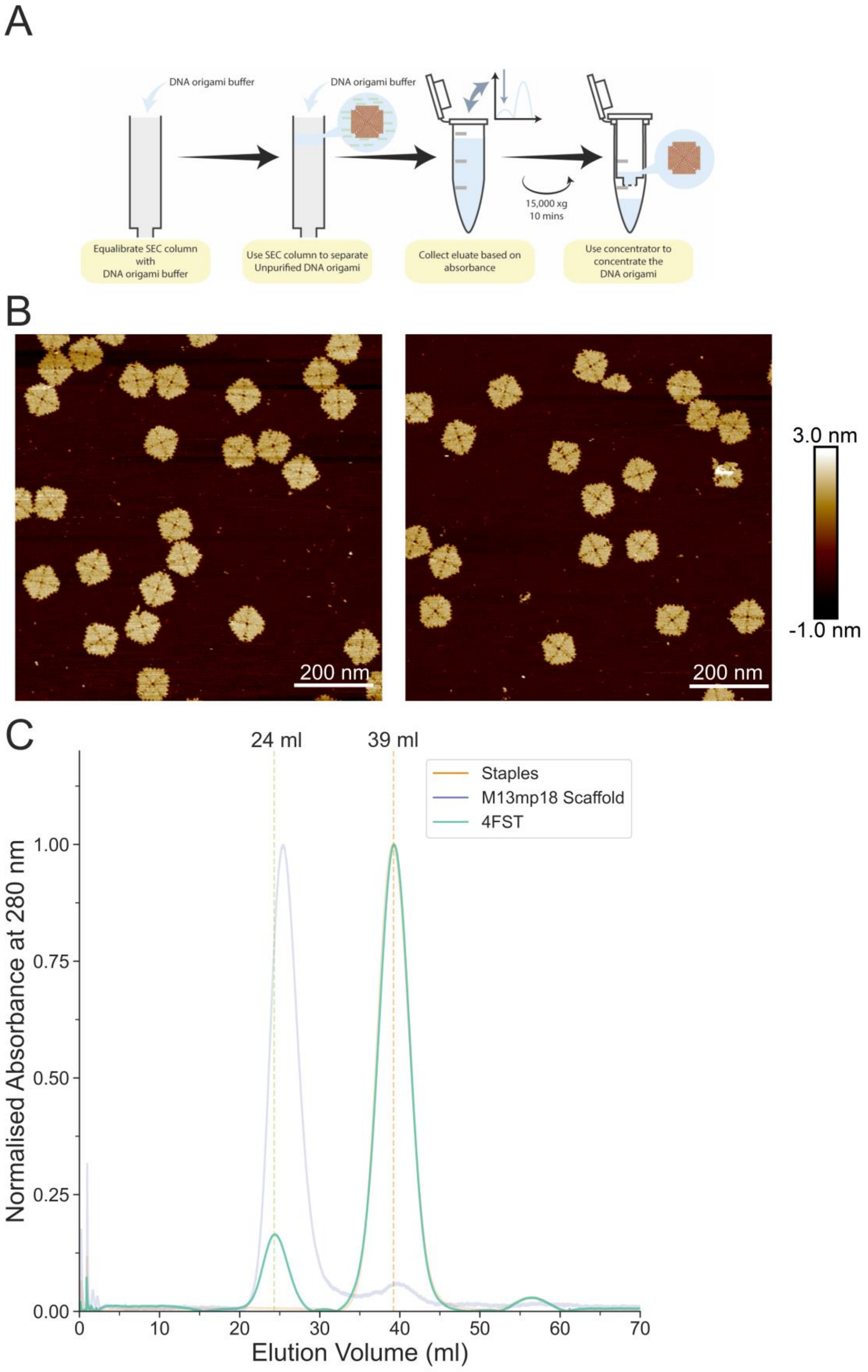

Supporting Figure S23. Purification of the 4FST DNA origami with the SEC method. (A) Schematic illustration of the procedure. (B) AFM images of the SEC purified 4FST DNA origami. (C) Overlapped elution profile of staples, M13mp18 scaffold, and the 4FST tile. The folded 4FST can be identified at 24 ml, the staples elute at 39 ml.

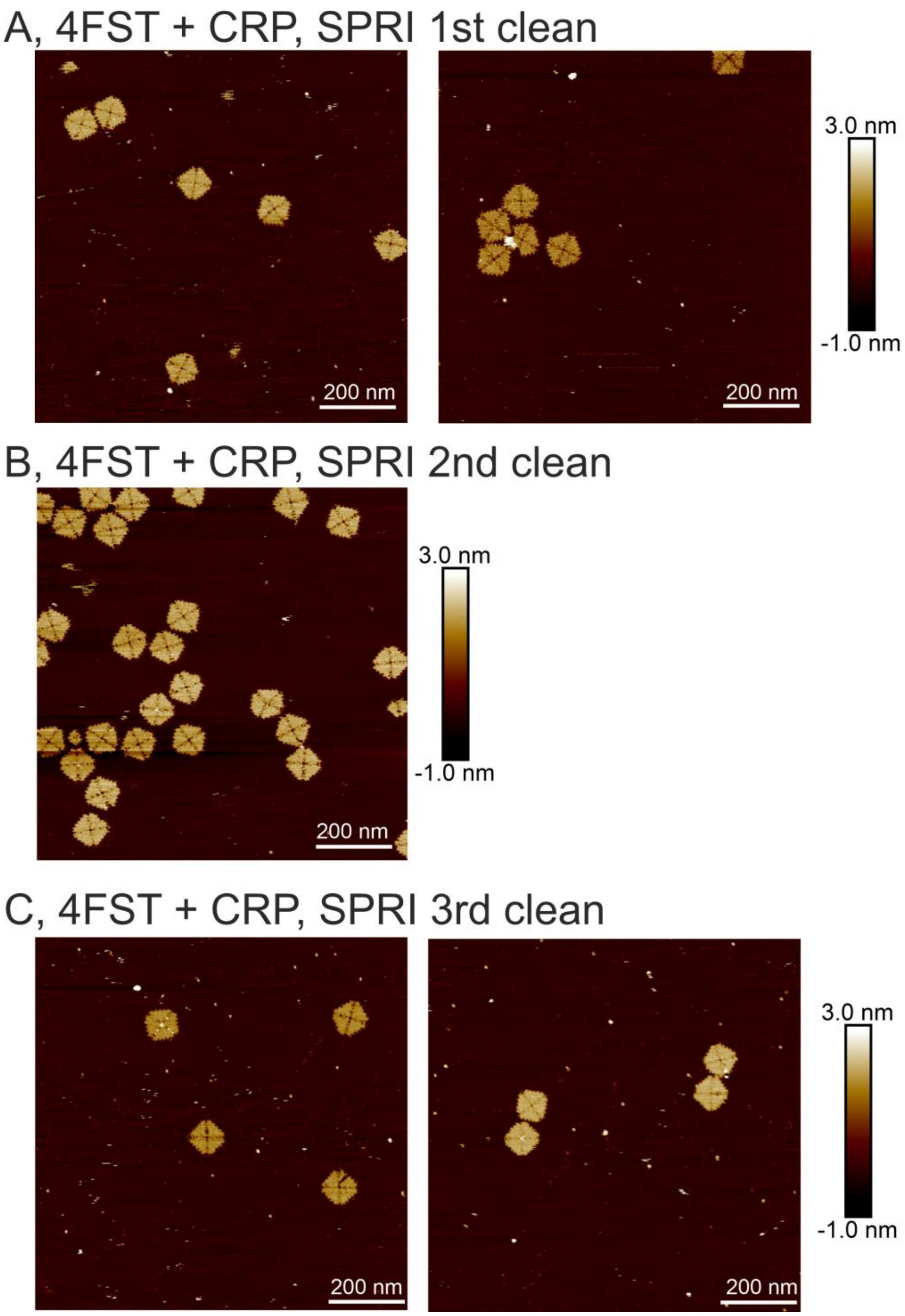

Supporting Figure S24. AFM images of the SPRI bead purification of the 4FST to remove excess CRP. (A) After one round of purification. (B) After two rounds of purification. (C) After the third round of purification.

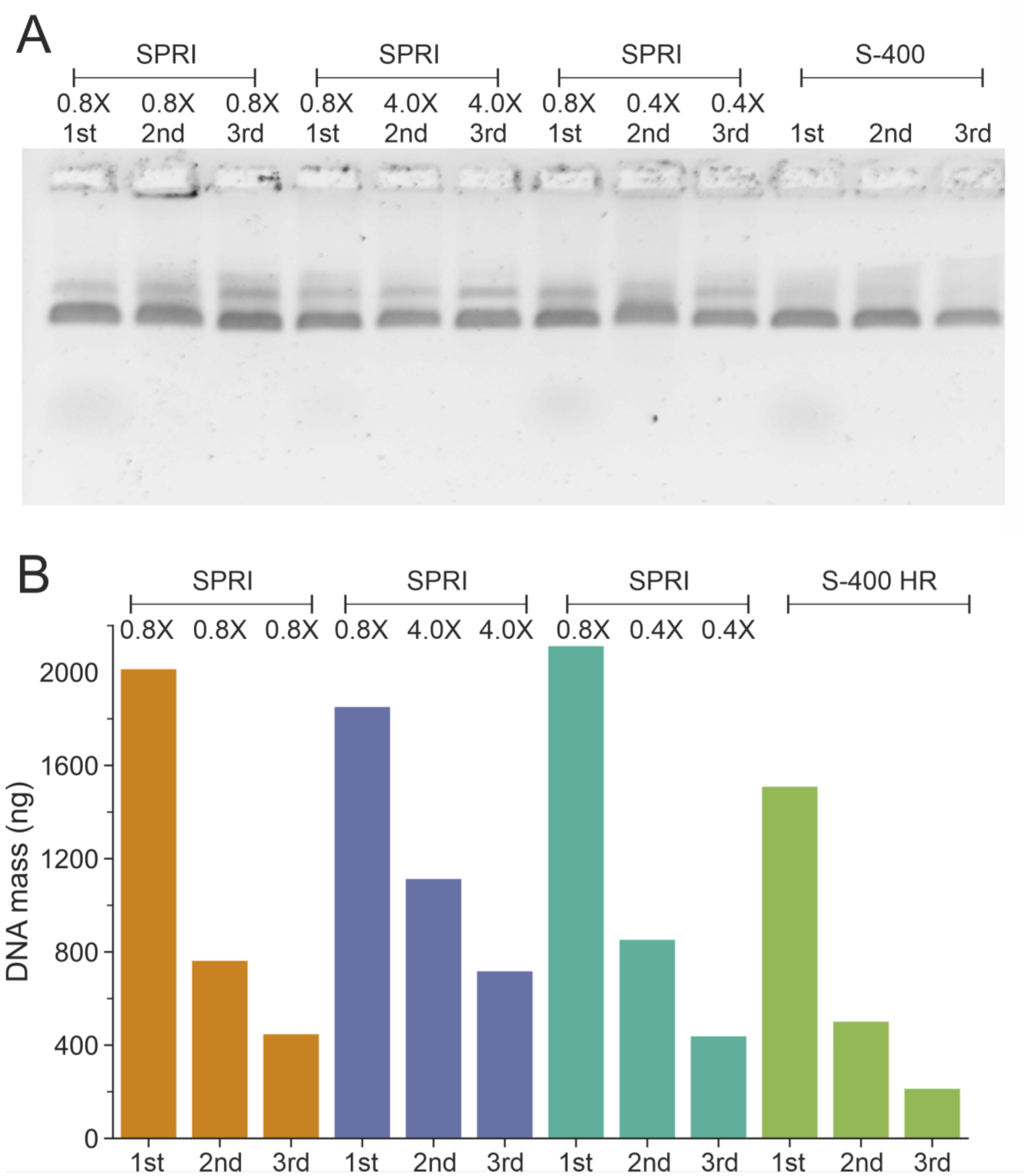

Supporting Figure S25. Successive rounds of purification. The 4FST DNA origamis were purified multiple times with different methods: SPRI bead purification at a volume ratio of 0.8X followed by two rounds of SPRI bead purification at volume rations of 0.8X, 4X, and 0.4X, respectively, and three times S-400 HR spin column. (A) Agarose gel electrophoresis image of the DNA origami samples after each round of purification. (B) The total DNA mass yield after each round of clean-up measured by A_{260} absorbance. Note, for this experiment a total of 80 µl origami reaction was used instead of the 40 µl used in other experiments. The left-most and right-most blocks show the same data as Figure 4D in the main text.

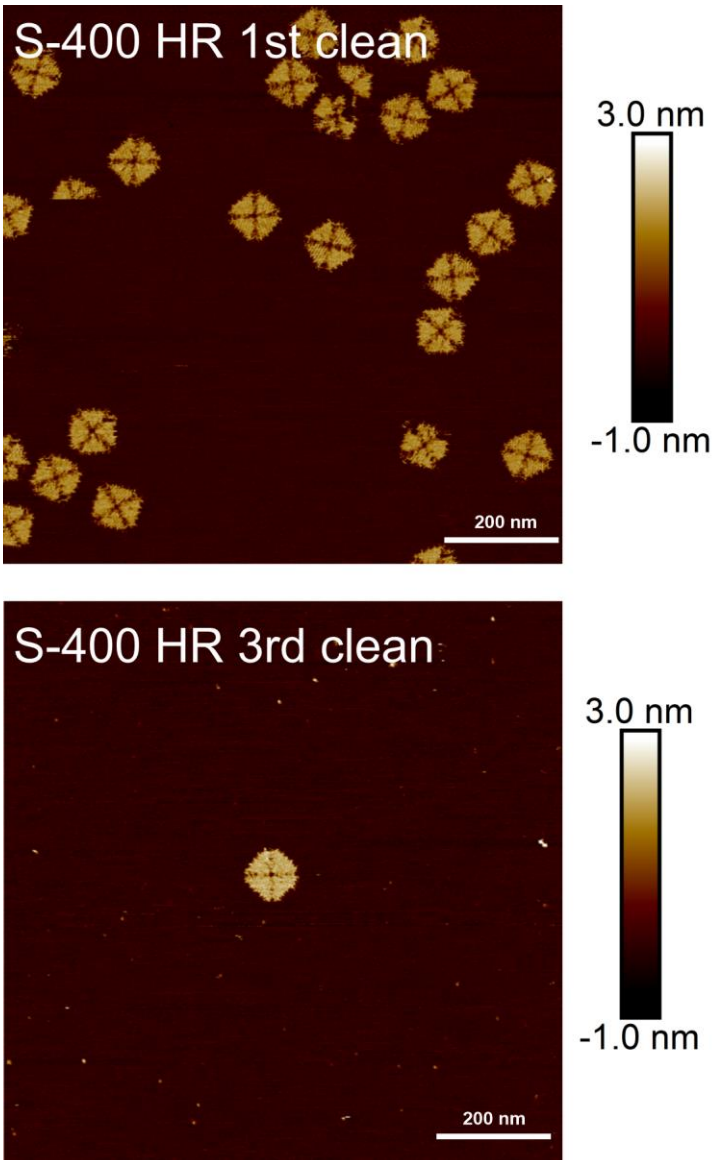

Supporting Figure S26. AFM images of the 4FST DNA origami after different rounds of S-400 HR spin column elution purification. (top) After the first round of purification, large numbers of DNA origami can be seen, with the large majority structurally intact. (bottom) After 3 rounds of S-400 HR clean up, the concentration of the DNA origami dropped significantly, and origamis were hard to find through AFM. However, the ones that were found were still structurally intact even after 3 rounds of purification.

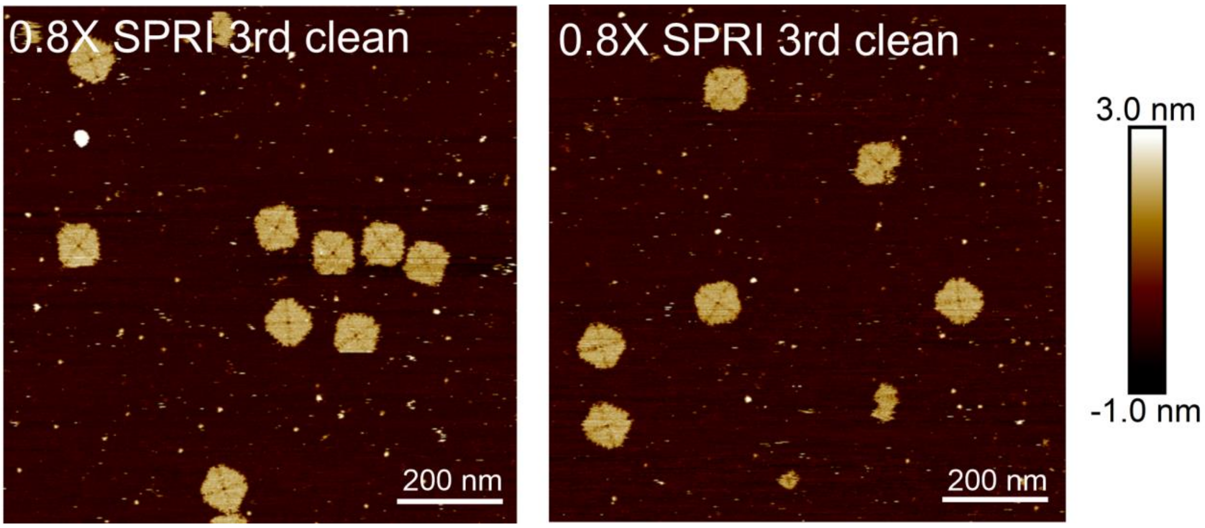

Supporting Figure S27. AFM images of the 4FST DNA origami after 3 rounds of SPRI beads purification. Two representative images of purified DNA origami. A significant number of DNA origami can be seen on each image, with most of them structurally intact even after 3 rounds of SPRI bead purification.

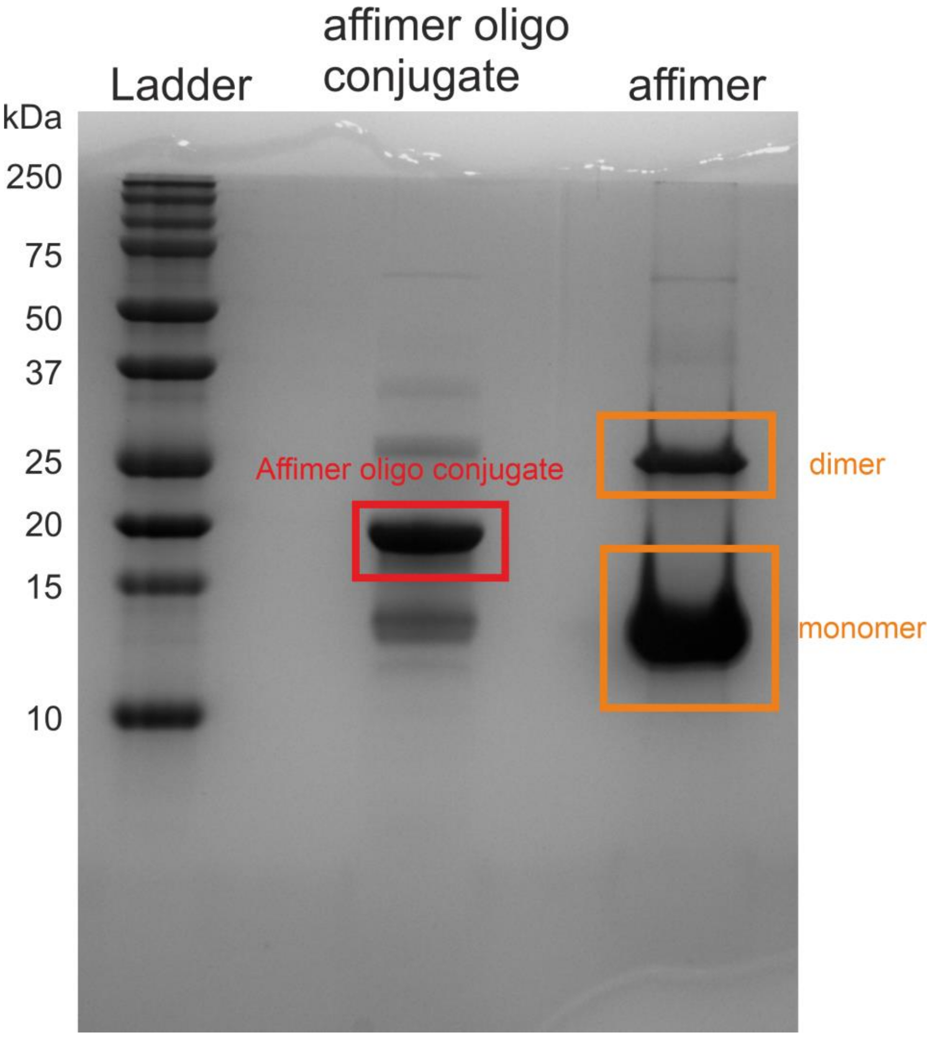

Supporting Figure S28. SDS-PAGE confirm the conjugation of the affimers to the oligos for hybridisation into the 4FSF origamis. SDS-PAGE was used to confirm the conjugation of the affimers to the oligos. Precision plus protein standard was used as protein ladder. A major band can be observed in the affimer oligo conjugate lane, the band migrated to approximately 20 kDa, which is in line with the calculated molecular weight of 19.0859 kDa for the affimer oligo conjugate. The affimer itself can form dimer due to disfulfide bonds between cysteine through oxidation, the monomeric affimer has a molecular weight of 12.06970 kDa and the dimer is 24.1394 kDa. The molecular weight of the 5' maleimide oligo is 7.0162 kDa.

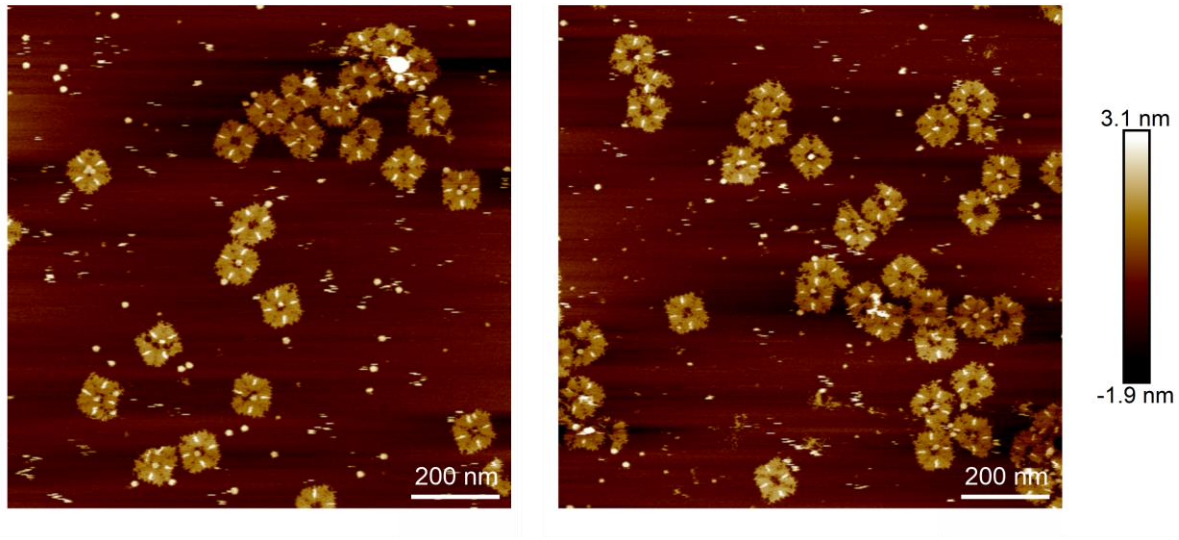

Supporting Figure S29. AFM images of the CRP bound CRP affimer functionalised 4FSF origami complex purified 0.8X SPRI bead volume ratio. Each 4FSF DNA origami was functionalised with 4 CRP affimer, the functionalisation was carried out via the thermal declump step, the affimer functionalised 4FSF was then mixed with CRP and then SPRI beads selection at 0.8X beads volume ratio was used again to remove the excessive CRP from the mixture prior to AFM imaging.

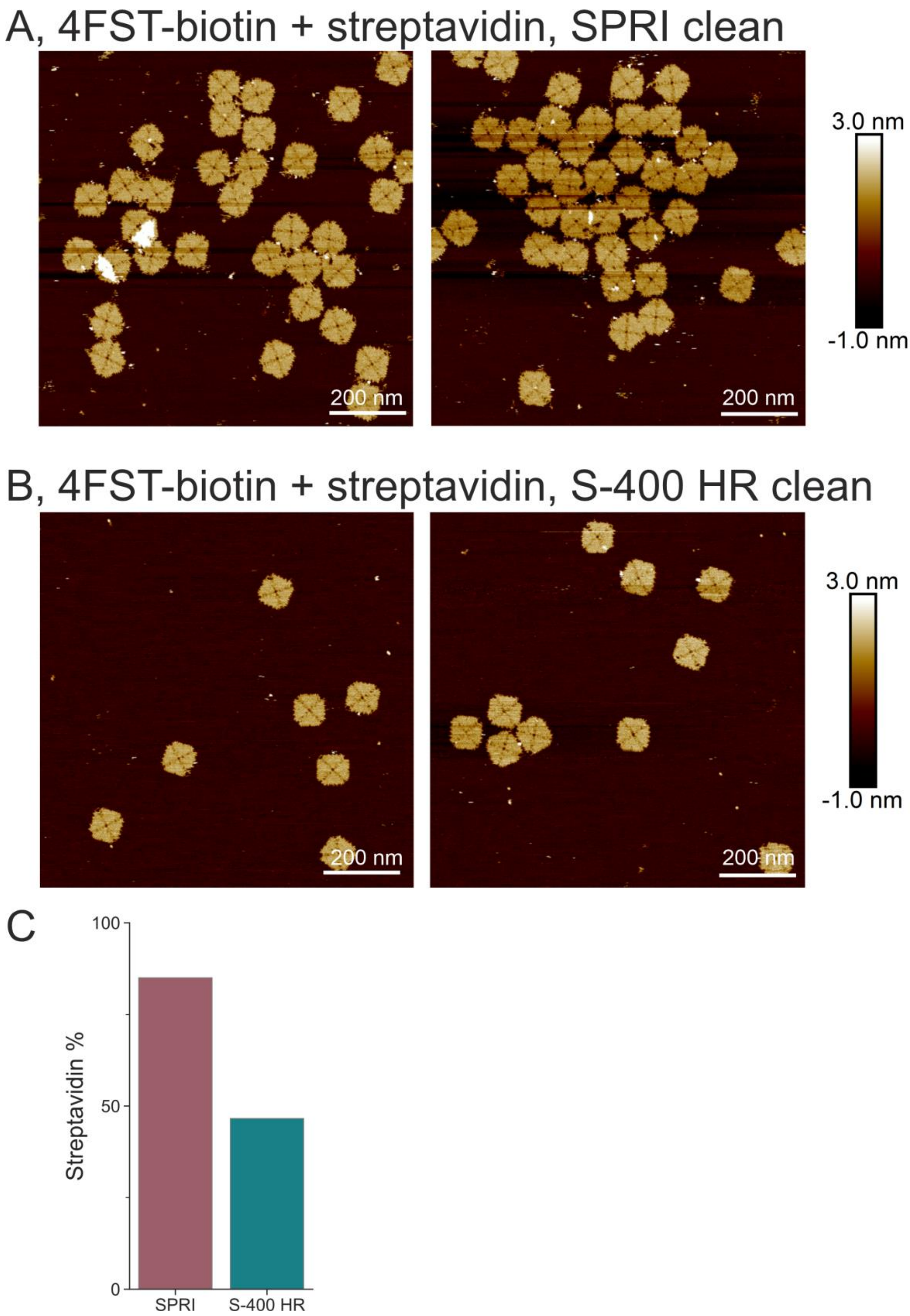

Supporting Figure S30. Comparison between the SPRI and S-400 HR spin columns on the streptavidin functionalised biotinylated 4FST DNA origami. Representative AFM images of the streptavidin-functionalised DNA origami after SPRI bead (A) and S-400 HR spin column (B) purification. (C) Bar graph showing the total yield of streptavidin functionalised 4FST origami purified using 0.8X volume ratio SPRI bead and S-400 HR spin columns.

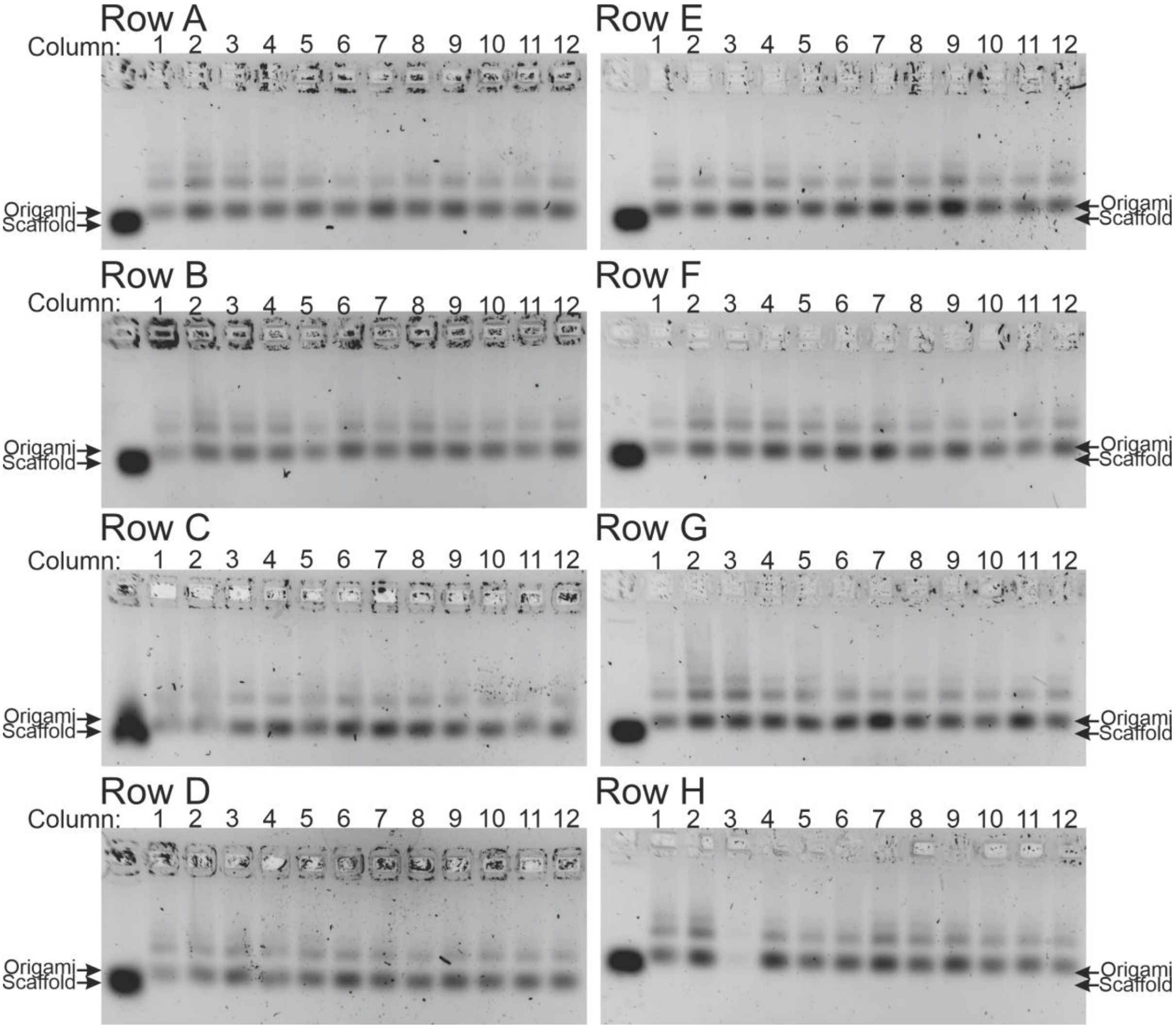

Supporting Figure S31. Agarose gel electrophoresis of all the samples in the 96 well plate of Prep 1 after automated SPRI bead purification of DNA origami using a liquid handling robot. A band indicating the presence of the 4FST DNA origami can be seen in 95 of the 96 wells. This purification run was performed with HighPrep SPRI beads. The third band appears above the dimer band missing in the main figure 5 was due to differences in gel stain time and exposure setting.

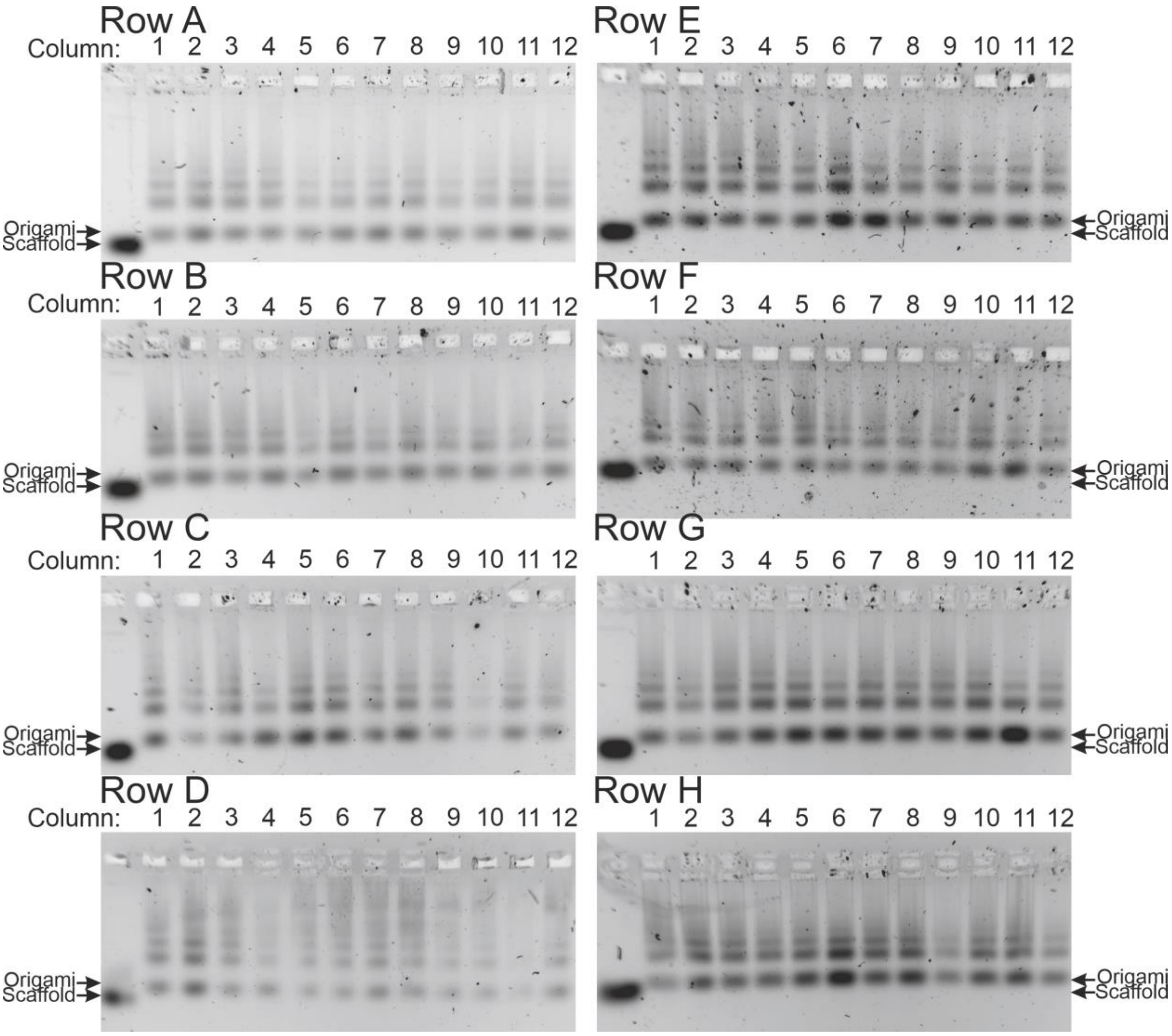

Supporting Figure S32. Agarose gel electrophoresis of all the samples in the 96 well plate of Prep 2 after automated SPRI bead purification of DNA origami using a liquid handling robot. A band indicating the presence of the 4FST DNA origami can be seen in 95 of the 96 wells. This purification run was performed with SPRIselect beads.

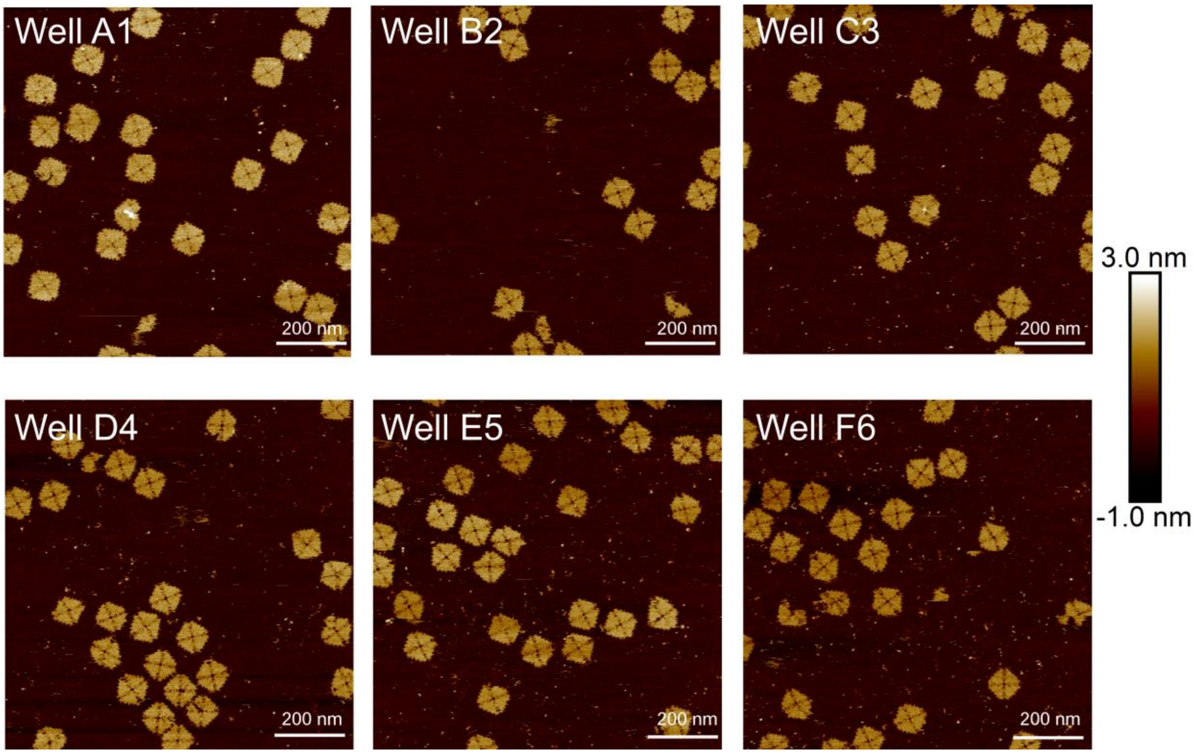

Supporting Figure S33. AFM images of the DNA origami purified robotically using SPRI beads. Six wells were selected (A1, B2, C3, D4, E5, F6) from the PCR plate of Prep 1. The purification was carried out with HighPrep SPRI beads using a liquid handling robot.

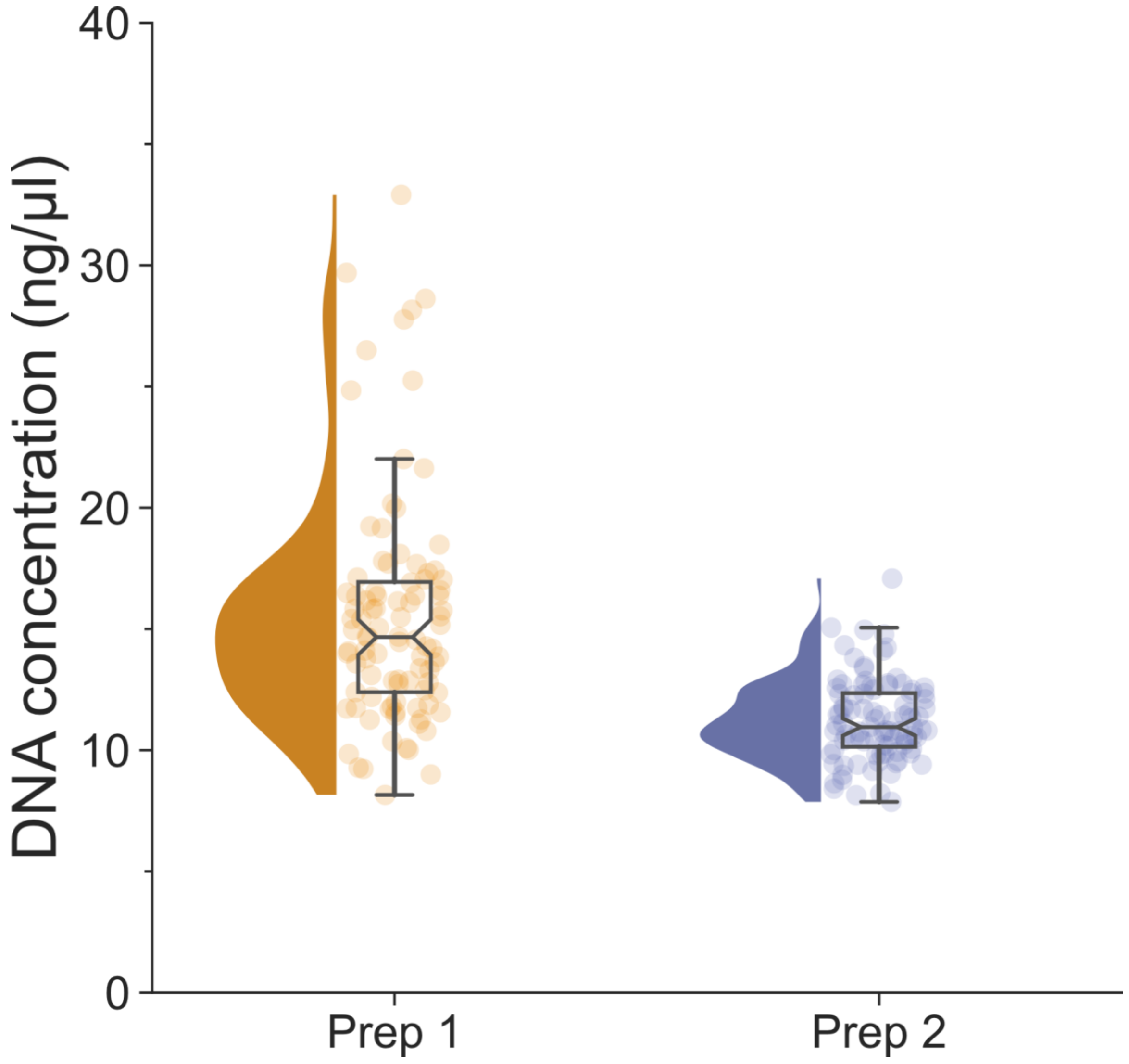

Supporting Figure S34. Amount of DNA origami in all samples of Prep 1 and 2 after automated SPRI bead purification via a liquid handling robot. The average concentration of purified DNA origami for Prep 1 is 15.56±0.48 ng/µl, and the average concentration of purified DNA origami for Prep 2 is 11.22±0.17 ng/µl. Errors represent the standard error of the mean.

Section 3: Supporting Instruction

These notes are aimed at providing some additional recommendations for adapting the usage of SPRI beads to purify DNA origami.

1. We recommend aliquoting the SPRI beads into tubes. Note, the beads tend to sediment relatively quick, so vigorously mixing prior to aliquoting will ensure consistent performance of the beads.
2. It is important to mix the beads well prior to use, the beads can be mixed vigorously by pipetting or vortexing.
3. Suppliers may change the SPRI bead solution components, therefore we recommend that an SPRI bead solution volume ratio screening should be performed for new batches, as the ratio presented here could differ.
4. For different structures, a ratio screen should be performed. In general, we found that ratio between 1.0X to 0.4X work well.
5. We recommend that the volume of the DNA origami should not be less than 20 µl. Due to the properties of SPRI beads, the volume can be adjusted to 40 µl without affecting the beads performance.
6. After the formation of the bead pellet, during the removal of the supernatant, some beads may be aspirated together with the supernatant. However, given the overall high yield of this method, the overall performance is not significantly impacted.
7. The last resuspension step, i.e. when we resuspending the solution in 40 µl to maintain the input and output volume consistency, this can be in any liquid volume, *i.e.* multiple folding reactions can be combined into one and eluted with smaller volume to concentrate the purified DNA origami.

References

1. Douglas, S. M.; Marblestone, A. H.; Teerapittayanon, S.; Vazquez, A.; Church, G. M.; Shih, W. M., *Nucleic Acids Research* **2009,** *37* (15), 5001-5006. DOI 10.1093/nar/gkp436.

2. Tikhomirov, G.; Petersen, P.; Qian, L., *Nature Nanotechnology* **2016,** *12* (3), 251-259. DOI 10.1038/nnano.2016.256.

3. Confederat, S.; Sandei, I.; Mohanan, G.; Wälti, C.; Actis, P., *Biophysical Journal* **2022,** *121* (24), 4882-4891. DOI 10.1016/j.bpj.2022.08.020.

4. Adamson, H.; Ajayi, M. O.; Campbell, E.; Brachi, E.; Tiede, C.; Tang, A. A.; Adams, T. L.; Ford, R.; Davidson, A.; Johnson, M.; McPherson, M. J.; Tomlinson, D. C.; Jeuken, L. J. C., *ACS Sensors* **2019,** *4* (11), 3014-3022. DOI 10.1021/acssensors.9b01574.

5. Raveendran, M.; Lee, A. J.; Sharma, R.; Wälti, C.; Actis, P., *Nature Communications* **2020,** *11* (1). DOI 10.1038/s41467-020-18132-1.

6. Stahl, E.; Martin, T. G.; Praetorius, F.; Dietz, H., *Angewandte Chemie International Edition* **2014,** *53* (47), 12735-12740. DOI 10.1002/anie.201405991.

7. Masukawa, M. K.; Sato, Y.; Yu, F.; Tsumoto, K.; Yoshikawa, K.; Takinoue, M., *ChemBioChem* **2022,** *23* (17). DOI 10.1002/cbic.202200240.

8. Lei, Y.; Mei, Z.; Chen, Y.; Deng, N.; Li, Y., *ChemNanoMat* **2022,** *8* (7). DOI 10.1002/cnma.202200161.

9. Lee, A. J.; Endo, M.; Hobbs, J. K.; Wälti, C., *ACS Nano* **2017,** *12* (1), 272-278. DOI 10.1021/acsnano.7b06208.
